# SGIP1 is involved in regulation of emotionality, mood, and nociception and modulates in vivo signalling of cannabinoid CB_1_ receptors

**DOI:** 10.1111/bph.15383

**Published:** 2021-02-27

**Authors:** Michaela Dvorakova, Agnieszka Kubik‐Zahorodna, Alex Straiker, Radislav Sedlacek, Alena Hajkova, Ken Mackie, Jaroslav Blahos

**Affiliations:** ^1^ Department of Molecular Pharmacology Institute of Molecular Genetics of the Czech Academy of Sciences Prague 4 Czech Republic; ^2^ Department of Psychological and Brain Sciences, Gill Center for Molecular Bioscience Indiana University Bloomington Indiana USA; ^3^ The Czech Center for Phenogenomics Institute of Molecular Genetics of the Czech Academy of Sciences Vestec Czech Republic

**Keywords:** anxiety, cannabinoid receptor 1, endocannabinoid system, GPCR, pain, tolerance

## Abstract

**Background and Purpose:**

Src homology 3‐domain growth factor receptor‐bound 2‐like endophilin interacting protein 1 (SGIP1) interacts with cannabinoid CB_1_ receptors. SGIP1 is abundantly and principally expressed within the nervous system. SGIP1 and CB_1_ receptors co‐localize in axons and presynaptic boutons. SGIP1 interferes with the internalization of activated CB_1_ receptors in transfected heterologous cells. Consequently, the transient association of CB_1_ receptors with β‐arrestin2 is enhanced and prolonged, and CB_1_ receptor‐mediated ERK1/2 signalling is decreased. Because of these actions, SGIP1 may modulate affect, anxiety, pain processing, and other physiological processes controlled by the endocannabinoid system (ECS).

**Experimental Approach:**

Using a battery of behavioural tests, we investigated the consequences of SGIP1 deletion in tasks regulated by the ECS in SGIP1 constitutive knockout (SGIP1^−/−^) mice.

**Key Results:**

In SGIP1^−/−^ mice, sensorimotor gating, exploratory levels, and working memory are unaltered. SGIP1^−/−^ mice have decreased anxiety‐like behaviours. Fear extinction to tone is facilitated in SGIP1^−/−^ females. Several cannabinoid tetrad behaviours are altered in the absence of SGIP1. SGIP1^−/−^ males exhibit abnormal behaviours on Δ^9^‐tetrahydrocannabinol withdrawal. SGIP1 deletion also reduces acute nociception, and SGIP1^−/−^ mice are more sensitive to analgesics.

**Conclusion and Implications:**

SGIP1 was detected as a novel protein associated with CB_1_ receptors, and profoundly modified CB_1_ receptor signalling. Genetic deletion of SGIP1 particularly affected behavioural tests of mood‐related assessment and the cannabinoid tetrad. SGIP1^−/−^ mice exhibit decreased nociception and augmented responses to CB_1_ receptor agonists and morphine. These in vivo findings suggest that SGIP1 is a novel modulator of CB_1_ receptor‐mediated behaviour.

**Abbreviations**
2‐AG: 2‐arachidonoylglycerol
AP‐3: adaptor protein 3
CME: clathrin‐mediated endocytosis
CRIP1: CB_1_ receptor interacting protein
ECS: endocannabinoid system
EPM: elevated plus maze
FC: fear conditioning
FCHO1/2: FCH/F‐BAR domain only protein 1 and 2
FE: fear extinction
GASP1: G‐protein‐associated sorting protein 1
LDB: light/dark box test
OF: open field
PPI: prepulse inhibition
SA: spontaneous alternation
SGIP1: Src homology 3‐domain growth factor receptor‐bound 2‐like endophilin interacting protein 1
THC: Δ^9^‐tetrahydrocannabinol
TIT: tail immersion test
TST: tail suspension test

**What is already known**

- The protein SGIP1 modulates signalling from activated CB_1_ receptors in vitro.
- SGIP1 interferes with the internalization of desensitized CB_1_ receptors.

**What this study adds**

- Genetic deletion of SGIP1 causes an anxiolytic‐like phenotype, reinforces cannabinoid tetrad behaviour and alters nociception.
- CB_1_ receptor agonists are more effective anti‐nociceptive agents in mice lacking SGIP1.

**What is the clinical significance**

- Activation of CB_1_ receptors is clearly involved in pain management.
- Regulation of CB_1_ receptors by SGIP1 may initiate approaches to enhanced anti‐nociception via CB_1_ receptors

## 1 INTRODUCTION

The endocannabinoid system (ECS) is involved in synaptic plasticity regulation with a wide range of physiological consequences. The cannabinoid CB_1_ receptor, a GPCR, is a central component of the ECS. GPCRs are regulated by universal mechanisms, including phosphorylation mediated by G‐protein coupled receptor kinases (GRKs) that trigger interactions with β‐arrestin and initiate endocytosis, via clathrin‐mediated endocytosis (CME) in the case of CB_1_ receptors. Besides signalling molecules common for all GPCRs, several other proteins have been reported to interact with CB_1_ receptors and to influence specific functions. The CB_1_ receptor interacting protein (CRIP1a) regulates CB_1_ receptor signalling and endocytosis, the adaptor protein 3 (AP‐3) affects processing and signalling of the internalized pool of CB_1_ receptors, and G‐protein‐associated sorting protein 1 (GASP1) controls lysosomal trafficking of down‐regulated CB_1_ receptors (Martini et al., 2007; Mascia et al., 2017; Niehaus et al., 2007; Rozenfeld & Devi, 2008).

The muniscin family of proteins comprise the Src homology 3‐domain growth factor receptor‐bound 2‐like endophilin interacting protein 1 (SGIP1), together with the ubiquitous FCH/F‐BAR domain only protein 1 and 2 (FCHO1/2) (Uezu et al., 2007). Muniscins interact with other molecules involved in CME, such as endophilin (Trevaskis et al., 2005), AP‐2 (Hollopeter et al., 2014), intersectin (Dergai et al., 2010), and Eps15 (Uezu et al., 2007). FCHO1/2 initiates plasma membrane invagination during the initiation of CME, while SGIP1 opposes this process. Hypothetically, the apparent difference between SGIP1 and FCFO1/2 domain organization within their N‐termini may explain their contrasting effects. FCHO1/2 proteins have their N‐terminal portion folded to form F‐Bar domains that are involved in the initiation of plasma membrane invagination during early stages of CME pit formation (Henne et al., 2007), while the N‐terminus of SGIP1 contains membrane phospholipid‐binding (MP) domain that has no sequence similarity to the F‐Bar motives and probably interacts with the plasma membrane in a way different from that of the F‐Bar domains of FCHO1/2. Most likely, the interaction of the MP domain with plasma membrane does not impose invagination of the membrane within the nascent pit formation.

SGIP1 is highly conserved across species, abundantly expressed in the nervous system, and enriched in compartments adjoining presynaptic boutons, in which it constitutes over 0.4% of protein content (Wilhelm et al., 2014). We did not detect SGIP1 when using our antibodies on immunoblots from peripheral tissues. In mice, SGIP1 and CB_1_ receptors have discernible overlapping expression patterns in most brain regions, including those involved in mood control, for example, in prefrontal cortex, striatum, and hippocampus, and nociception, namely, in the hypothalamus and other pain processing circuits (Lein et al., 2007). SGIP1 co‐immunoprecipitates with CB_1_ receptors from brain homogenates, and in neurons, the two molecules co‐localize in presynaptic compartments (Hajkova et al., 2016). One recognized physiological role of SGIP1 relates to regulation of energy homeostasis. Elevated levels of SGIP1 mRNA in the hypothalamus of the Israeli sand rat (*Psammomys obesus*) correlate with obesity of the animals held in captivity (Trevaskis et al., 2005), and genetic variations within the SGIP1 gene are associated with energy balance disturbances in humans (Cummings et al., 2012). Also, a possible association of mutations within the SGIP1 gene with neurological disorders has been reported in humans (Chwedorowicz et al., 2016). Interestingly, the ECS is involved in regulation of energy balance and in addiction. Thus, the CB_1_ receptors–SGIP1 relationship may be relevant here. On the other hand, genetic deletion of SGIP1 did not affect body weight implying that only overexpression of SGIP1 in the hypothalamus is associated with obesity.

CB_1_ receptors accumulate on axonal plasma membranes in cultured neurons, where they are substantially more stable than the receptors found on the neuronal soma (Coutts et al., 2001; Dudok et al., 2015; Leterrier et al., 2004; McDonald et al., 2007; Simon et al., 2013; Wu et al., 2008). Mikasova et al. used single‐quantum dot microscopy to study the properties of CB_1_ receptors on the surface of cultured cortical neurons and identified an immobile fraction of these receptors that remained on the plasma membrane in the vicinity of synapses for at least 30 min following agonist stimulation. Therefore, in the presynaptic compartments, a proportion of the CB_1_ receptors is resistant to agonist‐induced internalization and has low mobility (Mikasova et al., 2008).

Robust internalization follows the stimulation of CB_1_ receptors in transfected cells that lack SGIP1 (Daigle et al., 2008; Hsieh et al., 1999; Jin et al., 1999; Leterrier et al., 2004; Rinaldi‐Carmona et al., 1998). Co‐expression of SGIP1 with CB_1_ receptors interferes with agonist‐stimulated internalization of these receptors in this system (Hajkova et al., 2016). Functional consequences of SGIP1 association with CB_1_ receptors result from decreased down‐regulation of these receptors. Although G‐protein activation and termination of G‐protein‐mediated signalling are not affected by SGIP1, subsequent events that are facilitated by C‐tail phosphorylation in CB_1_ receptors and would result in CME, are markedly decreased by SGIP1. SGIP1 halts CME and the desensitized receptor remains on the cell surface.

Our current understanding suggests two broad pathways of GPCR signalling, one from the cell surface and a second signalling wave mediated by the internalized GPCR from intracellular compartments (Daaka et al., 1998). We proposed the following scheme for the effects of the relationship between SGIP1 and CB1R on events that follow the receptor desensitization. During CB_1_ receptor desensitization, arrestins interact with the phosphorylated receptors. The temporary association between phosphorylated CB_1_ receptors and arrestins terminates as the receptor is internalized. SGIP1 halts internalization of CB_1_ receptors. Therefore, the interaction of arrestin with the desensitized CB_1_ receptor persists longer in the presence of SGIP1. The consequence of stabilizing CB_1_ receptors at the cell surface by SGIP1 is that dissociation of arrestin from the receptor that follows internalization, occurs more slowly (Hajkova et al., 2016).

In our earlier study, we also observed that ERK1/2 signalling is decreased in the presence of SGIP1. This decrease is likely the consequence of a lack of the arrestin‐mediated ERK1/2 pathway activation from internalized CB_1_ receptors.

Therefore, SGIP1 adjusts CB_1_ receptor signalling in a biased manner; it does not influence CB_1_ receptor‐mediated G‐protein signalling at the cell surface, but it reduces ERK1/2 signalling from internalized CB_1_ receptors. For a schematic representation of these events, please see our earlier study (Figure 8, Hajkova et al., 2016).

We thus asked what would be the consequences of the SGIP1– CB_1_ receptor relationship in vivo. To gain insight into the physiological roles of SGIP1, we used a reverse genetic approach. For the current study, we developed mice with constitutively deleted SGIP1 for behavioural studies, assessing anxiety‐related behaviour, coping with unescapable situations, and we tested their acute nociception. We also studied the efficiency of CB_1_ receptor agonists and an opioid in mice lacking SGIP1, in nociception. Observations resulting from this study have the potential to improve pain management.

## 2 METHODS

### 2.1 Animals

All animal care and experimental procedures used in this study were in accordance to applicable laws, Guidelines of the National Institutes of Health on the Care and Use of Animals and to Directive 2010/63/EU. All animal models and experiments in this study were ethically reviewed and approved by the Institutional Animal Care and Use Committee of Indiana University or the Institute of Molecular Genetics, as appropriate for where the experiments were conducted. Animal studies are reported in compliance with the ARRIVE guidelines (Percie du Sert et al., 2020) and with the recommendations made by the British Journal of Pharmacology (Lilley et al., 2020).

Mice were bred on a C57Bl/NCrl background. SGIP1^−/−^ mice were generated in‐house starting with embryonic stem cells carrying the Sgip1^tm1b(EUCOMM)Hmgu^ allele obtained from the European Conditional Mouse Mutagenesis Program (EUCOMM). For more details about SGIP1^−/−^ mice production, see Supporting Information. For all behavioural studies, wild‐type (WT) and SGIP1^−/−^ mice were siblings of heterozygote parents of age 8 to 12 weeks. The mice were acclimated in the facility for 2 weeks prior to experiments. The animals were randomly distributed to the treatment groups. Testing was performed during the light phase of the circadian cycle. Mice were bred and group‐housed in accordance with animal welfare rules in a pathogen‐free facility with temperature 22 ± 2°C, 45% humidity, 12:12‐h light/dark cycle, and food and water ad libitum.

### 2.2 Spontaneous alternation

Exploration and distance travelled in the Y‐maze (Hughes, 2004) was recorded for a period of 5 min, and the percentage of arm alternations were calculated according to the equation %SA = (TA*100)/(TE − 2), where SA = spontaneous alternations, TA = total alternations made by animals, and TE = total entries to the arms, using software (Biobserve GmbH, Germany).

### 2.3 Startle reflex and prepulse inhibition

Animals were habituated to a holder in a soundproofed cabinet for 10 min (Med Associates Inc., USA). Animal testing composed of six repetitions, each included 10 sets of trials. The interval between the trials varied randomly from 10 to 20 s. Within each set of the trials, the mice were exposed to 10 repetitions of prepulse sound with defined intensities (0, 70, 77, 82, and 85 dB, 7 kHz), followed by a null (no sound), or startle sound (110 dB, 7 kHz). The delay between the prepulse and startle stimuli was 120 ms. The sound intensities of each prepulse and incidence of the startle (null or noise) alternated to ensure no repetitions of the same prepulse/startle pattern occur, and each of the patterns is presented only once within each set of the trials. The entire testing was performed on background noise (65 dB) (Yeomans & Frankland, 1995). Prepulse inhibition (PPI) is expressed as the percentage of decrease in startle reactivity amplitude caused by presentation of the prepulse (% PPI).

### 2.4 Open field, elevated plus maze, and light/dark box

The open field (OF) area was a 42 × 42 cm square, with a light intensity of 200 lux, virtually divided into peripheral and central zones (62% and 38% of the whole arena, respectively) (Choleris et al., 2001). Mice were placed individually in a corner and were observed for 20 min. We measured and analysed residence time in the central zone and the distance travelled in the whole arena (Biobserve GmbH).

The elevated plus maze (EPM) apparatus consisted of two closed and two open elevated arms, with a light intensity of 60 lux in the centre of the maze (Lister, 1987). The animal was placed in the centre and was left to explore the EPM for 5 min. The total time spent in open and closed arms and the centre and the total distance travelled were tracked as well as the number of visits of open and closed arms and the incidence of rearing (Biobserve GmbH).

The light/dark box (LDB) was divided into a light part (light intensity of 430 lux, 67% area) and an enclosed dark area (Bourin & Hascoet, 2003; Crawley & Goodwin, 1980). The animal was placed in the dark compartment and explored the arena for 5 min. The time spent in both sides of the box and the total distance travelled were analysed (Biobserve GmbH). The results are expressed as percentage of the total time spent in the distinct zones.

### 2.5 Tail suspension test

The animal was attached to an apparatus hook with tape and left suspended for 6 min to determine the duration of immobility (Porsolt et al., 1977). The immobility time was recorded and analysed using software BIO‐TST 4.0.2.1 (Bioseb, France).

### 2.6 Fear conditioning and fear extinction

Fear conditioning (FC) trial started with a 4‐min adaptation of the animal in the apparatus, after which the conditioned stimulus (CS; 20 s of 9‐kHz pure tone at 77 dB) and the unconditioned stimulus (US; foot shock; 1 s, 0.7‐mA scrambled current to the cage floor) followed (Stiedl et al., 1999). The US was presented with the termination of the CS. The dependent measure was freezing, defined as the absence of movement except for respiration. Animals were tested for contextual memory 24 h later in a novel context (new patterns on the walls, metal bars on the floor changed to a smooth plastic surface, and a different scent applied).

The fear extinction (FE) protocol was performed daily following the FC. The experiment was terminated when the freezing score did not evolve further (females 5, males 11 days). The tested animal was left to habituate for 1 min, and CS followed for 3 min. Freezing episodes during these times were automatically evaluated (Ugo Basile, Gemonio, Italy). The results are expressed as a percentage of the total time spent freezing during the CS.

### 2.7 Cannabinoid tetrad behavioural tests and THC withdrawal

The tetrad test included the ring tests, tail immersion test (TIT), body temperature measurements, and motor coordination assessment using a rotarod (Li et al., 2017) and was conducted in this order.

During the ring test, the mouse was placed on an elevated ring (6.35‐cm‐diameter wire ring suspended 16 cm above a flat platform) and recorded for 5 min to assess catalepsy (Pertwee, 1972). The TIT is described below. Body temperature was measured by rectal thermometer (Physitemp Instruments, USA). For the rotarod test, the mice were pre‐trained for 2 days prior to the experiment. The mice were placed on an accelerating rotarod (4–40 rpm), and the time spent on the rotating cylinder was recorded (Rahn et al., 2011). For chronic treatment with Δ^9^‐tetrahydrocannabinol (THC), mice received daily i.p. injections of 10 mg·kg^−1^ of THC and vehicle for 8 days. Behaviour was tested 60 min after THC injections on days 1, 4, and 8. Baseline data were obtained prior to each THC injection.

On day 9, the mice were injected once more with 10 mg·kg^−1^ THC or vehicle. The i.p. injection of a vehicle without any active substance was delivered 30 min later, and the inverse agonist rimonabant (SR141716) was applied 30 min later. Mouse behaviour was recorded for the entire period up to 30 min after rimonabant application. The incidence of withdrawal behaviours (head shakes, paw shakes, scratching and grooming, and jumping) was quantified as described previously (Cook et al., 1998; Morgan et al., 2014a).

### 2.8 Tail immersion test

The mice were gently restrained in a towel, and the tip of the animal's tail was immersed in 52°C water. Three measurements of latency to withdrawal with 30‐min intervals between individual measurements were recorded (Ramabadran et al., 1989). The cut‐off times for TIT when cannabinoid agonists THC and WIN 55,212‐5 were tested (10 s) or morphine (15 s) were adjusted in order to reach full effect with these compounds. The mice from this cohort were killed with the conclusion of the experiment.

The anti‐nociceptive effect of WIN 55,212‐5, THC, and morphine was evaluated as described (Morgan et al., 2014b; Nealon et al., 2019). Briefly, following baseline nociception assessment, the mice were injected with increasing doses of WIN 55,212‐5 (0, 0.3, 1, 3, and 10 mg·kg^−1^, i.p.), THC (0, 1, 3, 10, 30, and 50 mg·kg^−1^, i.p.), or morphine (0, 0.3, 1, 3, 10, and 30 mg·kg^−1^, i.p). The TIT was done 1 h after each injection, and succeeding injection with the higher dose followed immediately. For the rimonabant experiment, mice were injected daily for 3 days with 10 mg·kg^−1^ of rimonabant or vehicle for 3 days. The tail flick latencies were measured 30 and 60 min after each injection. Baseline responses were measured each day prior to the injections.

### 2.9 Order of testing

In tests with both sexes, the male and female cohorts were tested separately. The order of the behavioural tests was as follows: OF, SA, EPM, LDB, tail suspension test (TST), PPI, and TIT. For the FC and FE, we used naïve cohorts. For cannabinoid tetrad and withdrawal experiments, a new male cohort was used. Separate cohorts of naïve mice were used for each pharmacological experiment with rimonabant, WIN 55,212‐5, THC, and morphine.

### 2.10 Data and statistical analysis

The experimental procedures and data analysis were blinded to the experimenter and in cases of video analyses to the observer. The data and statistical analysis comply with the recommendations of the *British Journal of Pharmacology* on experimental design and analysis in pharmacology (Curtis et al., 2018). We used *F* test to analyse the homogeneity of sample variances employing R program, stats library. We did not detect violations of normality, or sphericity using R program (version 4), library moments in our data except the data in Figure 3i. Here, the analysis used the general linear model using Poisson link in R program (version 4), library stats (Team, 2020). We used qq plots to inspect normal distribution of residuals and calculated the correlation coefficient between observed residuals and theoretical residuals, R library Olsrr (Hebbali, 2020). We used log transformation for data which show abnormality in qq plot (Figures 2q, 3f,g, and 4e–h). Bonferroni post hoc test was applied only if *F* in ANOVA achieved *P* < .05 and there was no significant variance inhomogeneity. To analyse the ligand dose needed for 50% effect (ED_50_) in TIT, the curves were fitted as non‐linear regressions with variable slope (four parameters). The curves were constrained to 0 at the bottom and to 100 at the top. The ED_50_ values, the 95% confidence intervals, and Hill slopes were determined from the fit. Student's *t* tests, ANOVA, and non‐linear regression analyses were performed using GraphPad Prism version 8.0.1 for Windows (GraphPad Software, USA). The rimonabant experiment analysis was done by general linear model in R program (version 4), library stats. *P* < .05 was considered significant. In graphs, the error bars represent the SEM. Sample sizes for each cohort are specified in the Figures and legends. The tests used are specified in the Figure legends and the results of the statistical analysis are shown in Tables 1 and S1–S6. Table S7 summarizes methods of statistical analysis used specifically for each data set.

**TABLE 1:** Statistical analysis of dose–response data sets in Figure 4E–J using the ED_50_ values

	Sex	ED_50;_ (mg·kg^−1^)	95% CI	Hill slope	ED_50_	95% CI	Hill slope	*P* value
THC (mg·kg^−1^)	M	28.24	21.67–37.91	0.97	10.77	9.09–12.58	2.10	< .05
	F	31.20	24.63–40.13	1.32	30.67	23.31–37.23	2.40	> .05
WIN 55,212‐5 (mg·kg^−1^)	M	4.73	3.74–5.86	0.52	1.70	1.48–1.96	0.37	< .05
	F	18.11	12.63–38.23	0.26	3.20	2.71–3.83	0.45	< .05
Morphine (mg·kg^−1^)	M	4.86	3.88–6.14	0.44	2.18	1.70–2.76	0.36	< .05
	F	5.71	4.58–7.12	2.26	3.20	2.23–4.61	1.47	< .05

*Note*: The curves were fitted in GraphPad Prism 8.0.1. as non‐linear regressions with variable slope (four parameters). The curves were constrained to 0 at the bottom and 100 at the top. The ED50 values, the 95% confidence intervals, and Hill slopes were determined from the fit.. Abbreviations: CI, confidence interval; THC, Δ^9^‐tetrahydrocannabinol.

### 2.11 Materials

Chemicals were obtained from Sigma‐Aldrich (USA, Czech Republic) if not stated otherwise. The polymerase and immunoblotting reagents were purchased from Promega (USA, Czech Republic) and Thermo Fisher (USA, Czech Republic). WIN 55,212‐2 mesylate was obtained from Tocris (UK, Czech Republic). Δ^9^‐Tetrahydrocannabinol (THC) and morphine were kindly provided by Dr. Martin Kuchar (University of Chemistry and Technology, Prague, Czech Republic).

### 2.12 Nomenclature of targets and ligands

Key protein targets and ligands in this article are hyperlinked to corresponding entries in the IUPHAR/BPS Guide to PHARMACOLOGY (http://www.guidetopharmacology.org) and are permanently archived in the Concise Guide to PHARMACOLOGY 2019/20 (Alexander, Christopoulos et al., 2019; Alexander, Fabbro et al., 2019).

## 3 RESULTS

### 3.1 Deletion of the second exon from the SGIP1 gene leads to the loss of SGIP1 expression in mice

To generate the SGIP1^−/−^ mice, a novel DNA sequence with integrated Lox sites flanking the second exon was introduced by homologous recombination (Figure S1A). The mice lacking SGIP1 expression were generated using enzymic removal of this exon critical for protein expression. The second exon is composed of 64 base pairs. Its deletion results to a frameshift in the SGIP1 gene transcript, with stop codon insertion afterwards (Dickinson et al., 2016). The targeting and recombination events were detected by PCR from tail biopsies (Figure S1B). The SGIP1 protein was not expressed in the knockout (KO) mice, as confirmed on immunoblot from brain extracts (Figure S1C). The SGIP1^−/−^ mice were fertile, and no apparent abnormalities were observed, including growth and body weight (males: Figure S1D, females: Figure S1E).

### 3.2 SGIP1^−/−^ mice have normal working memory, exploration levels, and sensorimotor gating

**FIGURE 1:**
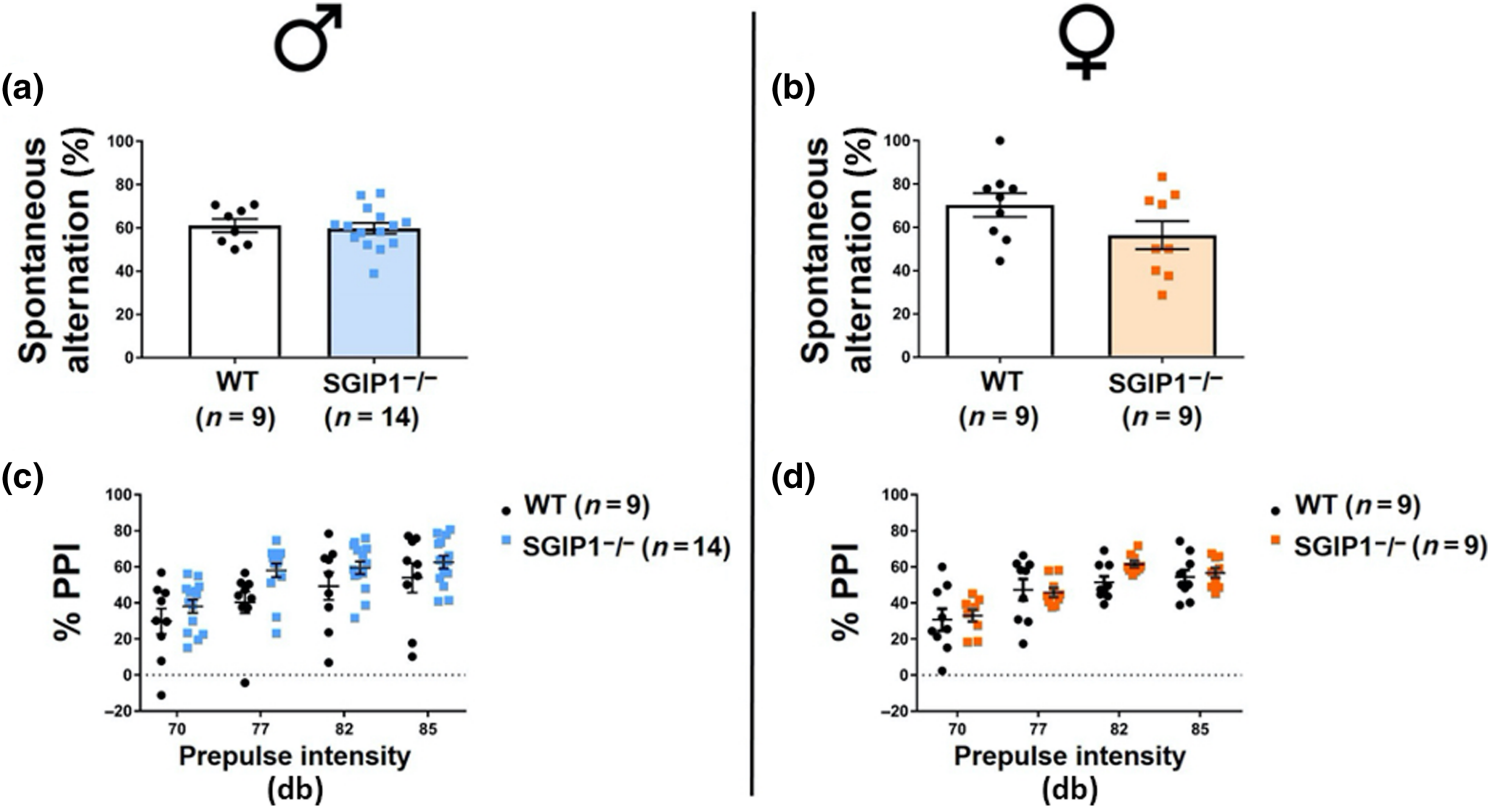
Cognitive functions and sensorimotor gating in mice is not affected by deletion of SGIP. Working memory was assessed as a percentage of spontaneous alterations in the Y‐maze. Both male and female SGIP1^−/−^ mice alternated the arms comparably to WT mice (males: a, females: b). Sensorimotor gating, measured as a percentage of prepulse inhibition (PPI), was not affected by the mouse genotype (males: c, females: d). The data were analysed by parametric *t* test (a, b) and by multiple *t* tests with the Holm–Sidak correction for multiple comparisons (c, d) and are presented as means ± SEM

Rodents show a tendency for alternations between the arms in the Y‐maze test. We examined short‐term working memory and exploration levels using this test. SGIP1^−/−^ mice showed no differences in the percentage of alternations between arms when compared with the WT groups (males: Figure 1a, females: Figure 1b).

Sensorimotor gating was evaluated by determination of PPI. No differences were observed in PPI between SGIP1^−/−^ and WT mice (males: Figure 1c, females: Figure 1d). For detailed statistical analysis, see Table S1.

### 3.3 SGIP1^−/−^ mice display signs of anxiolytic‐like phenotype and more vigorous response to unescapable situation

**FIGURE 2:**
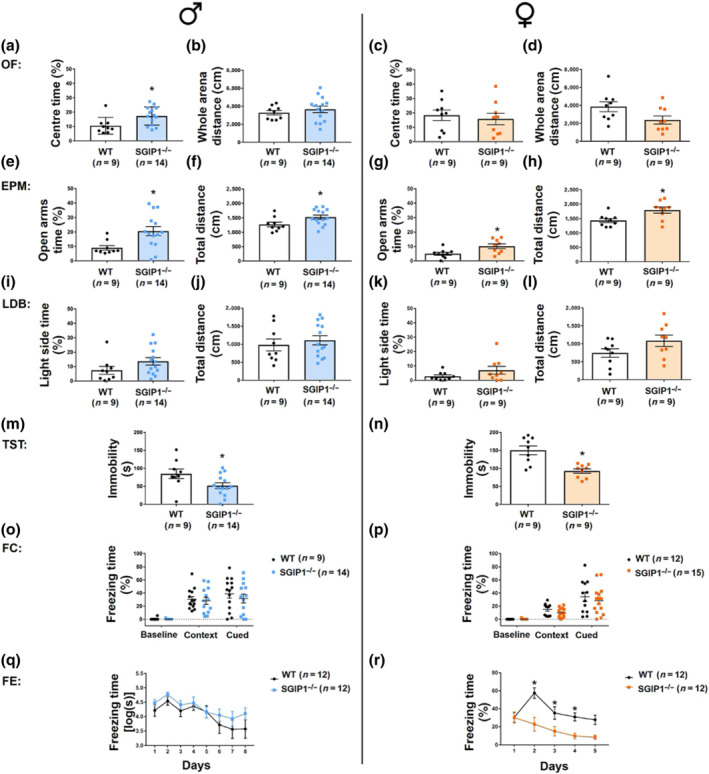
SGIP1^−/−^ mice show signs of altered emotionality. The exploratory and anxiety‐related behaviour of both male and female mice was tested in a set of behavioural paradigms: open field (OF) (a–d), elevated plus maze (EPM) (e–h), light/dark box (LDB) (i–l), tail suspension test (TST) (m, n), fear conditioning (FC) (o, p), and fear extinction (FE) (q, r). Male SGIP1^−/−^ mice spent more time in the centre of the open field (a) while walking comparable distances in the whole arena compared to WT littermates (b). We did not observe any differences in the time spent in the centre in female mice (c); however, there was a trend for the ambulatory behaviour expressed as distanced walked to be decreased in SGIP1^−/−^ females (d). Anxiety behaviour was further analysed in the EPM. Both male and female SGIP1^−/−^ animals spent significantly more time in the open arms (males: e, females: g) and walked longer distances (males: f, females: h). We did not detect any significant changes in the time spent in the light compartment of the LDB (males: i, females: k) or in the total distance walked between SGIP1^−/−^ and WT mice (males: j, females: l). To assess how the mice are coping with an unescapable situation, we employed the TST. Both male and female SGIP1^−/−^ mice spent significantly less time immobile in this test (males: m, females: n). We did not observe any difference in FC in male (o) or female (p) mice when we compared SGIP1^−/−^ to WT cohorts. The freezing time is expressed as a percentage of the total time. We did not observe any alteration in the FE to tone in SGIP1^−/−^ males (q). SGIP1^−/−^ females, on the other hand, show significantly decreased freezing on days 2–4 (r). Data were analysed by parametric *t* test (b–d, f–h, j, l–n) or by Mann–Whitney *U* test in the case of nonnormally distributed data (a, e, i, k). For the FC and FE, two‐way ANOVA with repeated measures was used for analysis (o–r). We used a logarithmic transformation of the data from male mice (q). The data are presented as means ± SEM. *P < .05, significantly different from WT; in ( r), *P < .05, significantly different from SGIP^‐/‐^

In the OF tests, SGIP1^−/−^ males spent 60% more time in the centre (Figure 2a), while distances walked in the entire arena were comparable with WT males (Figure 2b). We did not observe significant differences in the time spent in the centre for female mice (Figure 2c), and SGIP1^−/−^ females showed only a non‐significant trend for decreased distance walked (Figure 2d). The animals from all groups did not exhibit any unusual behaviours (e.g., explosive running and jumping). When not moving around, they rested, with occasional episodes of freezing, grooming, or rearing. We have compared additional indices—average speed, resting time, freezing behaviour, and rearing—and included the data in Figure S2. We observed higher incidence of rearing in SGIP1^−/−^ males, whereas SGIP1^−/−^ females did not show a significantly different incidence of the rearing behaviour. We also compared freezing time in the periphery and in the centre of the field to verify if animals reacted to anxiogenic centre by more frequent and longer freezing. There were no differences between the cohorts in freezing behaviour in either compartment. We also compared average speed and resting time in the centre and found that SGIP1^−/−^ females had lower average speed and increased resting time.

In the EPM tests, both male and female SGIP1^−/−^ animals spent significantly more time in the open arms (males: Figure 2e, females: Figure 2g) and walked longer total distances than the WT animals (males: Figure 2f, females: Figure 2h). We detected a 2.3‐fold and 2.1‐fold change in the open arms permanence time in male and female mice, respectively, when comparing SGIP1^−/−^ to WT mice. The number of visits into open and closed arms, as well as the incidence of rearing, was not significantly different in SGIP1^−/−^ mice (Figure S3A–F).

We conducted the LDB test with the same cohort of animals. We did not observe any significant differences between the WT and SGIP1^−/−^ mice regarding the time spent in the light compartment (males: Figure 2i, females: Figure 2k) nor in the total distance walked (males: Figure 2j, females: Figure 2l). In an independent testing with a naïve cohort of animals, the outcome of the LDB test was the same, with no significant differences between SGIP1^−/−^ and WT mice (data not shown).

To assess coping with an unescapable situation, as an estimate of depressive‐like behaviour, we used the TST. SGIP1^−/−^ mice of both sexes exhibited greater resilience than WT mice in an unescapable situation. In the TST, there is a 1.6‐fold change between SGIP1^−/−^ and WT both male and female mice. This indicates greater resilience in unescapable situation (Figure 2m,n). For detailed statistical analysis, see Table S2.

### 3.4 SGIP1^−/−^ mice have comparable levels of acute fear processing, but fear extinction varies between sexes

We examined fear conditioning of the aversive memory connected with context and a tone. The data are presented as percentage of the time spent freezing. Male SGIP1^−/−^ mice spent comparable time freezing as their WT littermates (Figure 2o). We also did not observe major differences in freezing of female SGIP1^−/−^ mice (Figure 2p). Extinction of the aversive memory (FE) connected with a tone occurred at a similar pace for both strains in male mice (Figure 2q). However, in female SGIP1^−/−^ mice, the extinction to tone was facilitated compared to WT female mice (Figure 2r). For detailed statistical analysis, see Table S2.

### 3.5 Cannabinoid tetrad tests reveal alterations in SGIP1^−/−^ mice

We compared SGIP1^−/−^ and WT male mice behaviour in the cannabinoid tetrad tests (catalepsy, anti‐nociception, hypothermia, and suppression of motor coordination) to study initial responses and development of tolerance to THC treatment (10 mg·kg^−1^·day^−1^, i.p., in 9‐ to 10‐week‐old male mice). We injected the mice for 8 days and tested their behaviour on days 1, 4, and 8 (Martin et al., 1991).

**FIGURE 3:**
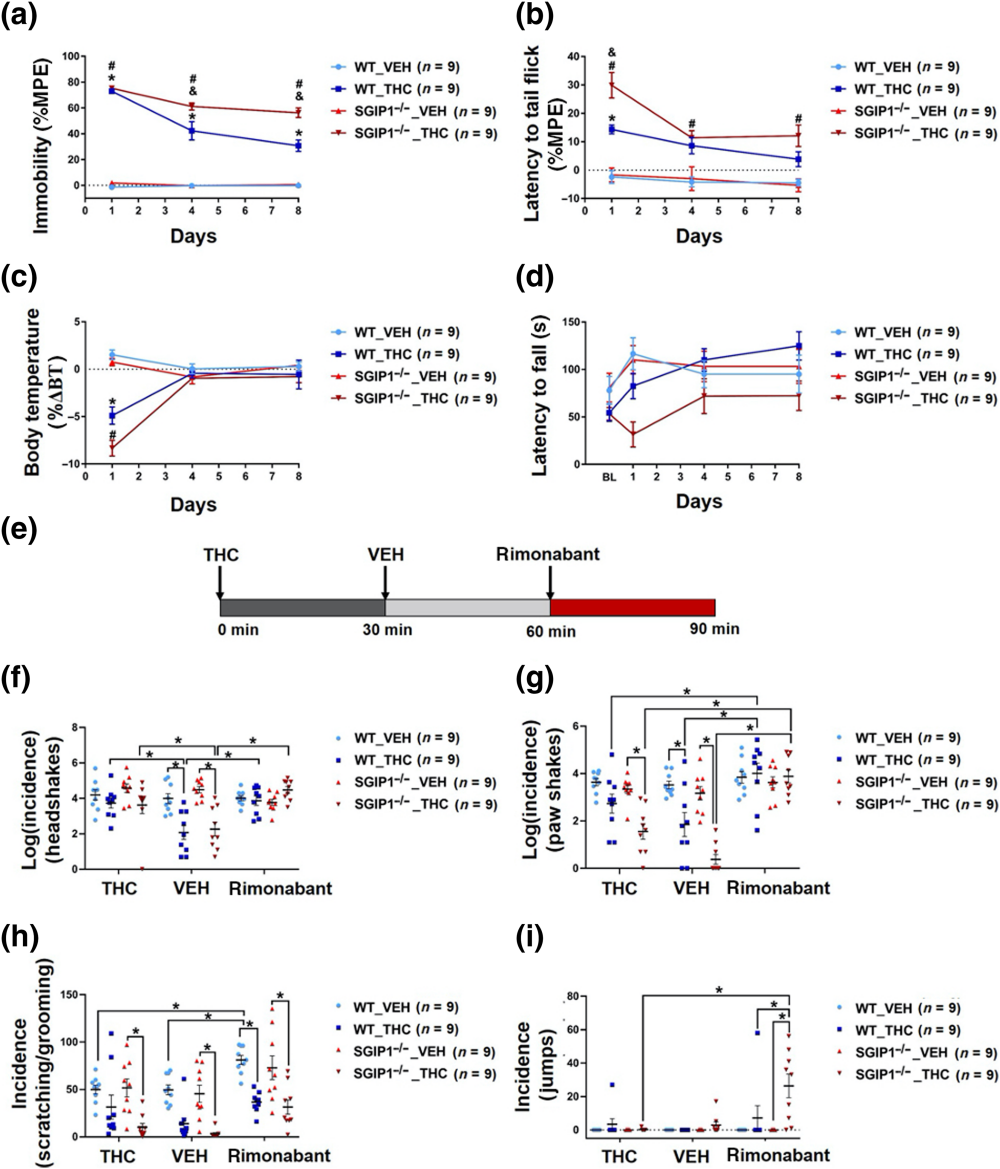
SGIP1 absence affects anti‐nociception, catalepsy, and body temperature in the cannabinoid tetrad test, and THC withdrawal triggers jumping. Males were injected with 10 mg·kg^−1^, i.p., of THC daily for 8 days and tested on days 1, 4, and 8. The baseline response was measured each day prior to the THC injection. The data are plotted as percentage of the maximal possible efficacy (%MPE) in the ring test and tail immersion test, as percentage of body temperature change (%ΔBT) for the body temperature measurements and as raw values in the rotarod test. In the ring test, the baseline responses and responses after the first drug delivery were comparable for animals from both groups. On days 4 and 8, the SGIP1^−/−^ mice were more cataleptic than the WT mice (a). In the TIT, the latencies were prolonged in the SGIP1^−/−^ mice group even prior to the THC injection. On the first day, the THC injection doubled the latency in SGIP1^−/−^ mice compared to WT mice; however, this effect was not maintained on days 4 and 8 (b). WT and SGIP1^−/−^ mice temperature lowered significantly after the first THC injection. The effect of genotype in this test was detected by ANOVA on day 1 (c). The rotarod test did not reveal significant effect of genotype (d). To study THC withdrawal symptoms, after nine consecutive days of THC injections (10 mg·kg^−1^), a CB1R inverse agonist rimonabant was used. Thirty minutes after the last THC injection, the mice were injected with a vehicle and after another 30 min with rimonabant (10 mg·kg^−1^) (e). We studied the incidence of THC withdrawal signs: headshakes (f), paw shakes (g), and scratching/grooming (h). There were no relevant differences between WT and SGIP1^−/−^ mice in the manifestations of headshakes, paw shakes, and scratching/grooming; however, after rimonabant application, SGIP1^−/−^ mice jumped more frequently (i). The data from the tetrad tests and withdrawal were analysed by three‐way ANOVA with repeated measures (a–d, f–h) followed by Bonferroni post hoc test. For the withdrawal data without normal distribution, we used logarithmic transformation (f, g), and for analysis of incidence of jumping, the general model with the Poisson link in program R was used (i). Data are presented as means ± SEM. In (a‐c),*P < .05, significant effect of THC in WT mice; ^#^ *P* < .05, significant effect of THC in SGIP1^−/−^ mice; ^&^ *P* < .05, SGIP1^−/−^ (THC treated) significantly different from WT (THC treated); in (f‐i), *P < .05, significantly different as indicated

To assess catalepsy, we used the ring test. While the duration of catalepsy was comparable for animals from both groups after the first drug doses, on the fourth and eighth days, the THC‐treated SGIP1^−/−^ mice were cataleptic for a longer time than the WT mice. Neither group of sham animals showed differences in these tests (Figure 3a). The THC significantly prolonged the latencies in TIT in SGIP1^−/−^ mice compared to WT mice on day 1 (fold change 1.6.); on day 4, the difference was significantly less pronounced (fold change 1.4); and on day 8, the SGIP1^−/−^ mice again displayed 1.8‐fold higher antinociceptive effect upon chronic delivery of THC than the WT mice (Figure 3b). The hypothermic effect evoked by acute THC treatment was greater in SGIP1^−/−^ mice than in WT mice (Figure 3c). In the rotarod tests, the effect of genotype between SGIP1^−/−^ and WT mice was not significant prior to and following the treatments (Figure 3d). For detailed statistical analysis, see Table S3.

### 3.6 THC withdrawal of SGIP1^−/−^ mice

WT and SGIP1^−/−^ males were given 10 mg·kg^−1^·day^−1^ of THC for 9 days. On the ninth day, the mice were injected with 10 mg·kg^−1^ THC, followed by vehicle 30 min later, and another 30 min later, 10 mg·kg^−1^ rimonabant was applied (Figure 3e). Following the rimonabant application after chronic delivery of THC, we monitored behaviours associated with withdrawal (Figure 3f–h). We did not observe an increased incidence of headshakes or scratching/grooming after the rimonabant injection in THC‐pretreated WT and SGIP1^−/−^ mice (Figure 3f,h). We detected a higher incidence of paw shakes in THC‐pretreated WT and SGIP1^−/−^ mice after the rimonabant injection; however, there was no significant difference between the two genotypes (Figure 3g). Interestingly, in SGIP1^−/−^, the withdrawal was expressed as intense jumping (Figure 3i). The jumps were manifested as straight leaps in the air with a strong charging from all four paws (see Movie S1). For detailed statistical analysis, see Table S4.

### 3.7 Delayed nociception and enhanced sensitivity to CB_1_ receptor agonists and morphine in SGIP1^−/−^ mice

**FIGURE 4:**
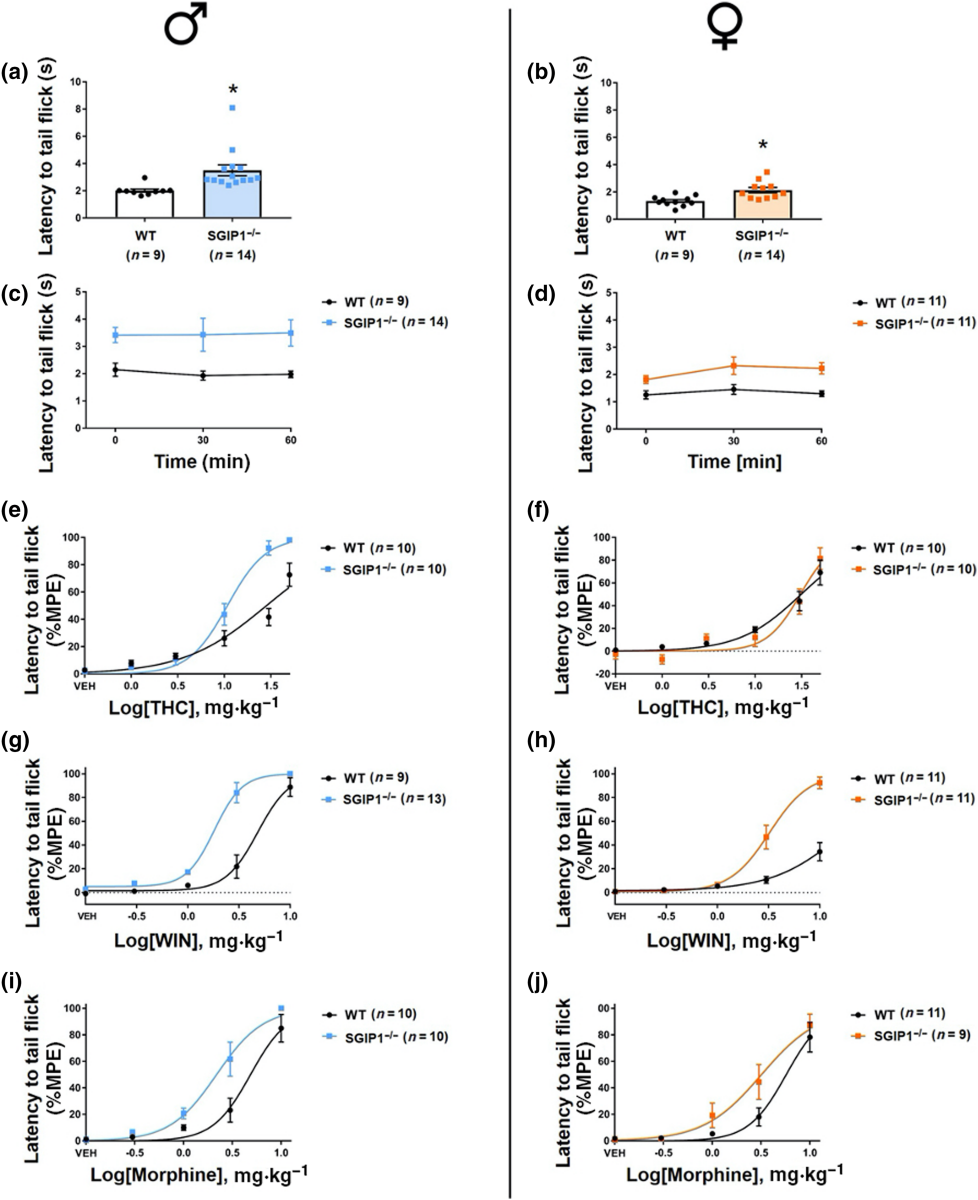
SGIP1^−/−^ mice have an anti‐nociceptive phenotype. To assess anti‐nociception, we used the tail immersion test (TIT). The tip of the mouse's tail was immersed into a water bath of 52°C, and the latency to flick the tail was measured. We measured the latencies in three trials with 30‐min inter‐trial intervals and averaged the responses. Both sexes of SGIP1^−/−^ mice have prolonged latencies to flick the tail (males: a, females: b). Lower panel graphs show that there is no significant difference between the individual trials, averaged in (a) and (b) (males: c, females: d). To assess the anti‐nociceptive effect of CB_1_ receptor agonists or an opioid, mice were i.p. injected with increasing doses of THC (0, 1, 3, 10, 30, and 50 mg·kg^−1^), WIN 55,212‐2 (WIN; 0, 0.3, 1, 3, and 10 mg·kg^−1^), or morphine (0, 0.3, 1, 3, 10, and 30 mg·kg^−1^), and after 1 h, the tail flick was measured. THC dose–response curve is shifted to the left in male SGIP1^−/−^ mice (e), but both female SGIP1^−/−^ and WT mice showed similar responses in the TIT (f). The WIN dose–response curve is shifted to the left, which indicates decreased nociception in SGIP1^−/−^ mice of both sexes compared to WT controls (males: g, females: h). We observed a similar leftward shift in male and female SGIP1^−/−^ mice in the morphine dose–response experiment (i). For the dose–response experiments, the data are plotted as percentage of the maximal possible efficacy (%MPE). The data were analysed by parametric *t* test (b), Mann–Whitney *U* test (a), and two‐way ANOVA with repeated measures (c, d). The calculated ED_50_ values of the curves from panels (e)–(j) are shown in Table 1. Data are presented as means ± SEM. *P < .05, significantly different from WT

We detected different responses to heat stimuli in WT and KO animals. In the TIT, the latency to react to the heat stimuli was significantly prolonged in male and female SGIP1^−/−^ mice (Figure 4a; females: Figure 4b). There is a 1.7‐fold and 1.5‐fold change in latencies of SGIP1^−/−^ compared to WT male and female mice, respectively. We examined nociception in three trials with 30 min inter‐trial intervals and plotted individual values as well. The latencies to tail flick do not change over time (males: Figure 4c, females: Figure 4d). For detailed statistical analysis, see Table S5.

To further study the effect of SGIP1 deletion on CB_1_ receptor‐mediated anti‐nociception, we injected the mice with increasing doses of THC (0, 1, 3, 10, 30, and 50 mg·kg^−1^, i.p.) in TIT to obtain a dose–response relationship. The THC dose‐response curve shifts leftward in male SGIP1^−/−^ mice (Figure 4e), whereas in female mice, there was no significant difference in the THC‐induced anti‐nociception (Figure 4f). Next, we tested the anti‐nociceptive effect of WIN 55,212‐5 (0, 0.3, 1, 3, and 10 mg·kg^−1^, i.p.) in the TIT experiments. The dose‐dependent anti‐nociceptive effects of WIN 55,212‐5 were augmented in SGIP1^−/−^ mice of both sexes when compared to the WT mice (males: Figure 4g, females: Figure 4h). Lastly, we tested the anti‐nociceptive effects of morphine (0, 0.3, 1, 3, 10, and 30 mg·kg^−1^, i.p.) by measuring the latencies to tail flick, as above. There was a leftward shift of the morphine dose–responses in male and female SGIP1^−/−^ mice (Figure 4i,j). For THC treatments of the WT and SGIP1^−/−^ male cohorts, the ED_50_ values and 95% confidence intervals are stated in Table 1. We analysed further the dose–responses for WIN 55,212‐5 and morphine and detected distinct patterns of cooperativity for the two ligands with the SGIP1 deletion. When comparing these drug effects in the male cohorts, the effects of WIN 55,212‐5 appeared to be synergistically enhanced in SGIP1^−/−^ males compared with WT males. However, the increased sensitivity to morphine, in the TIT dose‐response assays, was suggestive of additive‐like effect in the SGIP1^‐/‐^ mice (Figure S4).

### 3.8 Transient effects of the CB_1_ receptor antagonist on nociception in SGIP1^−/−^ mice

**FIGURE 5:**
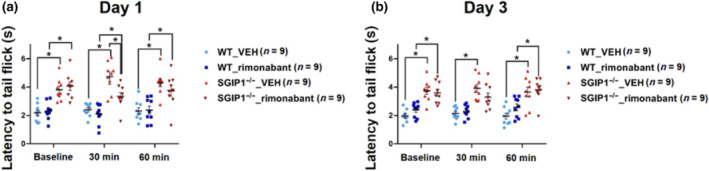
CB_1_ receptor antagonist temporarily alters nociception of SGIP1^−/−^ mice. To assess the effect of the CB_1_ receptor antagonist on nociception, we injected the mice with 10 mg·kg^−1^·day^−1^ of rimonabant or vehicle for 3 days and measured their tail flick latencies on days 1 and 3 each day 30 and 60 min after injections. On day 1, 30 min after the injections, we observed significantly decreased latencies in SGIP1^−/−^ mice treated with rimonabant compared to SGIP1^−/−^ mice injected with vehicle (a). We continued the treatments for three consecutive days and measured the responses on day 3. The TIT responses following 30 min after the treatments with rimonabant between the WT and SGIP1^−/−^ mice were not significantly different (b). The data were analysed by general linear model using program R and are presented as means ± SEM. *P < .05 significantly different as indicated

We examined the responses of WT and SGIP1^−/−^ mice to the known CB_1_ receptor antagonist rimonabant in the TIT. We continued the treatments for three consecutive days and measured the responses to the drug on days 1 and 3. On day 1, we observed significantly decreased latencies to tail flick in SGIP1^−/−^ mice treated with rimonabant, compared to vehicle‐treated SGIP1^−/−^ mice, 30 min after the injections (Figure 5a). The initial differences did not persist when the latencies were measured 60 min after the injections (Figure 5a). The TIT responses following the 3‐day treatment with rimonabant between the WT and SGIP1^−/−^ mice were significantly different, 60 min after the treatment, but not 30 min after the injections (Figure 5b). For detailed statistical analysis, see Table S6.

## 4 DISCUSSION

### 4.1 Sensorimotor processing and working memory in SGIP1^−/−^ mice

SGIP1 null mice were used in a reverse genetic approach to investigate the role of SGIP1 in vivo in the behavioural tests. SGIP1^−/−^ mice have phenotypes restricted to particular tasks, namely, in tests that examined aspects of mood‐related behaviours and nociception, while other modalities, including their mobility, exploratory drive, and working memory, remained intact. These results indicate that the development of the nervous system in SGIP1^−/−^ mice was not profoundly affected. Both male and female SGIP1^−/−^ mice have similar exploratory drives and mobility in the Y‐maze test. We also verified the integrity of sensorimotor gating in the SGIP1^−/−^ mice. The PPI test assesses sensorimotor processing by measuring both transmissions of afferent sensory information and motor responses following efferent signalling. Sensorimotor processing and working memory are not altered in SGIP1^−/−^ mice. As performance in both the Y‐maze and PPI tasks was normal, we exclude altered exploratory drives or impaired sensorimotor gating as causes of the observed differences in further behavioural examinations.

### 4.2 Mood‐related behaviour and emotionality in SGIP1^−/−^ mice

OF, EPM, and LDB tests are based on the conflict between the drive to explore a new environment and the avoidance of aversive light areas. SGIP1^−/−^ males spent more time in the centre of the OF arena than WT males, while female SGIP1^−/−^ and WT mice had comparable times in each section of the arena. We examined the levels of anxiety in tests assessing anxiety‐like behaviours. In the EPM test, both male and female SGIP1^−/−^ mice spent more time in the open arms and walked longer distances in the maze than WT mice. We did not observe any significant differences between the two groups in the LDB test, even when we tested an autonomous cohort with a group of animals that were not previously exposed to any testing. SGIP1^−/−^ mice exhibited greater resilience in an unescapable situation, as assessed in the TST.

The ECS is involved in controlling mood, processing of fear, and adaptive handling of stressful situations (see Lutz et al., 2015; Mechoulam & Parker, 2013; Micale et al., 2013; Morena et al., 2016). Our results from the EPM and OF experiments imply an anxiolytic‐like phenotype for SGIP1^−/−^ mice, with variability between sexes. These data are compatible with previous reports (Fattore & Fratta, 2010). The anxiolytic‐like phenotype of SGIP1^−/−^ and higher resistance to unescapable situation are in accordance with pharmacological studies demonstrating anxiolytic‐like and antidepressive‐like effects of enhanced endocannabinoid transmission by blocking their metabolic degradation in rodents (Bortolato et al., 2007; Danandeh et al., 2018). Anxiety and depression have a high rate of co‐morbidity. Previous investigations have noted overlapping molecular pathophysiological mechanisms of both modalities. Perhaps, further studies will elucidate if SGIP1 engages the same pathways leading to two distinctive phenotypes.

Further studies of the intracellular signalling by the activated, internalized CB_1_ receptors will benefit from the availability of the SGIP1^−/−^ mice. These behavioural studies (OF, EPM, and LDB) will require cohorts of mice chronically treated with perorally supplied rimonabant. This would be required to the detect consequences of different rates of internalization of the activated CB_1_ receptors in WT and SGIP1^−/−^ mice. Such treatments will avoid the undesired carry‐over effects of the stress, induced by handling of the animals, leading to ECS activation.

In the TIT experiment with rimonabant treatments for 3 days, we observed residual differences between the WT and SGP1^−/−^ cohorts. Therefore, more extended treatment, or higher doses of the compound, will be required to prevent lasting signalling by the ECS and consequences of the activation of CB_1_ receptors, prior to the testing. However, such treatment will not eliminate the effects of crosstalk of ECS with other systems involved in nociception. Animals treated chronically with rimonabant will be an essential control. The results of the testing of this cohort will have to be compared to behaviours of mice that will be exposed to acute stress prior to the delivery of rimonabant. A comparison of outcomes of such testing may allow us to estimate the role of the internalized pool of CB_1_ receptors in these tasks.

Extinction of fear memories is modulated by the ECS (Lutz et al., 2015; Marsicano et al., 2002). Our current study revealed a strong sex‐related difference in FE. In the FC tests, both in a context and as a response to a cue, the results were comparable between SGIP1^−/−^ and WT mice for both sexes. Extinction of the aversive memories was comparable for SGIP1^−/−^ and WT males, but there was a significantly more efficient FE in SGIP1^−/−^ female mice compared to WT females. Differences between sexes are known for this process (see Velasco et al., 2019). For instance, anatomical differences between male and female mice in densities of CB_1_ receptors in the hippocampus have been correlated with sex‐dependent differences in FE (Lopez‐Gallardo et al., 2012; Reich et al., 2009). As discussed above, our previous study revealed that SGIP1 has a negative impact on CB_1_ receptor‐mediated ERK1/2 signalling levels (Hajkova et al., 2016). Differences between ERK1/2 levels of signalling were correlated with FE variabilities (Matsuda et al., 2015). The SGIP1^−/−^ mice are therefore suitable to study the outcomes of this signalling pathway on behaviour, in future studies. Based on our results, we would conclude that SGIP1 can regulate anxiety levels under specific contexts, possibly via modulation of CB_1_ receptor signalling.

### 4.3 Responses to acute and chronic THC treatment

We examined the acute responses and tolerance progression to THC in the cannabinoid tetrad tests. After initial THC treatments, SGIP1^−/−^ and WT mice displayed similar levels of catalepsy but the SGIP1^−/−^ mice developed a tolerance to THC‐induced catalepsy at a significantly slower rate. SGIP1^−/−^ mice also exhibited enhanced THC antinociception with a significant effect on the first day of dosing that decayed over the next 7 days of repeated drug delivery at a rate similar to WT mice. Similarly, THC‐induced hypothermia was augmented in SGIP1^−/−^ mice, and they progressively developed tolerance to this effect. In the rotarod tests, WT and SGIP1^−/−^ animals performed comparably (Figure 3d). For detailed statistical analysis, see Table S3.

Following the chronic treatment with THC, rimonabant was applied, to provide a model of withdrawal, resulting in abnormal behaviours in the SGIP1^−/−^ mice, with intense and persistent jumping. Interestingly, similar jumping has also been reported upon morphine withdrawal (Francis & Schneider, 1971) and is decreased in mice lacking CB_1_ receptors (CB1R^‐/‐^; Ledent et al., 1999). In our previous investigations in vitro, SGIP1 markedly affected CB_1_ receptor‐mediated β‐arrestin2 signalling (Hajkova et al., 2016). β‐Arrestin2 association with the CB_1_ receptor occurs upon phosphorylation of serine or threonine residues within the receptor's carboxy‐terminal. Mice with a double mutation of two critical serine residues to alanine in the CB_1_ receptor (S426A, S430A) have enhanced sensitivity to THC (Morgan et al., 2014a). Thus, mice with the mutant CB_1_ receptor (S426A, S430A) and SGIP1^−/−^ mice have an overlapping phenotype in this regard. Another apparent similarity between these strains is in their withdrawal after chronic THC treatments. In both cases, withdrawal resulted in jumping. Crosstalk between the ECS and other signalling pathways, namely, the opioid system, may explain this observation on a molecular level (Canals & Milligan, 2008; also reviewed in Robledo et al., 2008).

### 4.4 Nociception and pharmacological evaluation of CB_1_ receptor agonists and morphine in SGIP1^−/−^ mice

Activation of CB_1_ receptors is known to blunt reactions to painful stimuli (Hasanein et al., 2007; Mascarenhas et al., 2017; Woodhams et al., 2017) and we found SGIP1^−/−^ mice to have prolonged reaction latencies in the TIT, compared with WT animals. This was observed upon both single and repeated tests. As a part of the tetrad tests, we also studied the effect of THC on nociception. SGIP1^−/−^ mice had elevated latency in the TIT before THC treatment and enhanced sensitivity to the drug. Also, after daily THC administration for 8 days, tolerance developed more slowly in SGIP1^−/−^ males. Moreover, in male SGIP1^−/−^ mice, there was an enhanced anti‐nociception to THC and WIN 55,212‐5 in the TIT. This effect was particularly noticeable in the delayed responses upon increasing doses of the CB_1_ receptor agonists (Figure 4e,g, respectively). Re‐analyses of the dose‐response for WIN 55,212‐5 suggest that there was synergy between WIN 55,212‐5 and SGIP1 deletion, in terms of the anti‐nociceptive effects (Figure S4). However, morphine, in the TIT, interacted differently with SGIP1 deletion. While morphine‐induced anti‐nociception was still enhanced in SGIP1^−/−^ mice, this interaction was weaker than that with the CB_1_ receptor agonist and was more consistent with an additive type of interaction (Figure S4).

In our earlier in vitro study, we tested the effect of SGIP1 on CB_1_ receptor signalling using two agonists, 2‐arachidonoylglycerol (2‐AG) and WIN 55,212‐5 (Hajkova et al., 2016). Following activation by either of these agonists, G‐protein‐mediated signalling by CB_1_ receptors was unaffected by the presence or absence of SGIP1 (Figure 6b, Hajkova et al., 2016). Arrestin recruitment, and activation of ERK1/2 signalling stimulated by WIN 55,212‐5 was, in general, greater than that elicited by 2‐AG and was markedly depressed in the presence of SGIP1, compared with the results of 2‐AG application (Hajkova et al., 2016) (Figure 7a,b). It would seem that when there is a low level of arrestin recruitment to theCB_1_ receptor and less signalling via ERK1/2, the effects of SGIP1 are also modest. When there is robust arrestin recruitment to the receptor and higher levels of ERK1/2 pathway activation, SGIP1 plays a more dominant role (Hajkova et al., 2016). Further studies will be necessary to find out if the in vitro observations correlate with the current in vivo findings.

The inverse agonist rimonabant has been shown to elicit hyperalgesia in TIT in several previous reports (Costa & Colleoni, 1999; Meng et al., 1998; Richardson et al., 1998), while in other studies, the authors did not detect any effect on nociception in mice (Compton et al., 1996; Rinaldi‐Carmona et al., 1994). In our experiments, pre‐treatment with the CB_1_ receptor antagonist rimonabant resulted in transiently increased nociception in SGIP1^−/−^ males, 30 min after the injections, but this effect did not persist after 1 h. Interestingly, on day 3 of the treatment, rimonabant‐treated SGIP1^−/−^ cohort responses were not significantly different from the WT cohort. The inverse agonist possibly influences the cognitive aspect of nociception in this test, as the stress of injection might play a role in activating the ECS, an effect that might be significant at early, but not later post‐injection time points (Woodhams et al., 2017). In accord with previous reports, we also detected sex‐dependent variation of the ECS effects on nociception (Fattore & Fratta, 2010). We propose that SGIP1 may well be a novel regulator of CB_1_ receptor‐mediated anti‐nociception.

### 4.5 The phenotype of SGIP1^−/−^ mice coincides with behaviour detected following genetical and pharmacological manipulations of ECS

The results of the behavioural tests described in previous studies, in which the ECS was manipulated chemically or genetically, may be related to the present study. Global deletion of CB_1_ receptors (CB1R^‐/‐^) also resulted in a modified exploratory phenotype, hypoactivity and anxiety‐like behaviour, if the CB1R^−/−^ mice were subjected to highly aversive conditions (Zimmer et al., 1999). In another study, moderate doses of CB_1_ receptor agonists evoked anxiolytic effects (while higher doses lead to the opposite) (Rey et al., 2012). The anxiolytic phenotype that we observed in the tests with SGIP1^−/−^ mice parallels the situation with moderately up‐regulated tone within the ECS. This is in accordance with our hypothesis about the SGIP1 effect on CB_1_ receptor signalling.

Modification of the endocannabinoid degradation and synthesis pathways also influences behaviour. Increasing anandamide levels via chemical inhibition of its catabolic enzyme fatty acid amide hydrolase (FAAH) (Kathuria et al., 2003) or the deletion of FAAH (Moreira et al., 2008) resulted in phenotypes with behavioural aberrations including decreased anxiety‐like behaviour, as we observed in the present tests with SGIP1^−/−^ mice. On the other hand, global deletion of DAG lipase (DAGLα), the enzyme primarily involved in neuronal 2‐AG synthesis, results in increased levels of anxiety‐like behaviour (Jenniches et al., 2016; Shonesy et al., 2014). We conclude that our results of the behavioural testing of SGIP1^−/−^ mice are comparable with the phenotype in which the ECS signalling had been modified. Altered CB_1_ receptor signalling in the SGIP1^−/−^ mice may thus be imposed on adjacent signalling cascades. Mice lacking β‐arrestin2 also exhibited enhanced acute responses to THC and altered tolerance following repeated THC treatment (Breivogel et al., 2008; Nguyen et al., 2012). As discussed above, SGIP1 influences the association of CB_1_ receptors with β‐arrestin2 and signalling mediated by this relationship. Also, genetic disruption of GASP1 results in reduced tolerance to cannabinoid‐mediated antinociception in the TIT in mice (Martini et al., 2010).

The results from studies with mouse strains with manipulated levels of β‐arrestins or GASP1 closely resemble our observations using SGIP1^−/−^ mice and further supports our hypothesis that SGIP1 affects behaviour by the modification of CB_1_ receptor signalling.

In summary, SGIP1^−/−^ mice have specific variations in a discrete subset of behavioural tests and nociception. This resonates with observations from similar studies that used mouse models with mild enhancements of neuronal ECS signalling. Also, SGIP1^−/−^ mice responses to CB_1_ receptor agonists are affected by SGIP1 deletion in several tests. Together with our previously reported findings that SGIP1 and CB_1_ receptors interact with functional consequences and the data presented here, we propose that the SGIP1^−/−^ mouse phenotype may be a consequence of the alteration of CB_1_ receptor signalling and that SGIP1 regulates the function of CB_1_ receptors in vivo.

There are, however, several unresolved questions to answer. For example, does SGIP1 influence CB_1_ receptor signalling uniformly throughout the nervous system, or is this effect confined to certain neuronal populations? As not all CB_1_ receptor‐associated physiological effects were affected equally by SGIP1 deletion, we hypothesize that only particular neuronal subtypes, or circuits, may be selectively modulated in SGIP1^−/−^ mice or that the phenotype is apparent only when reaching certain thresholds of system engagement. Also, potential interactions between SGIP1 and other receptors or signalling systems await further investigation.

## AUTHOR CONTRIBUTIONS

A.H. and R.S. were involved in the SGIP1^−/−^ mice production. M.D., A.K.Z., and J.B. carried out the experiments and analysed the data. J.B., M.D., A.K.Z., K.M., A.S., and R.S. contributed to the writing of the manuscript. J.B. and K.M. supervised the project.

## CONFLICT OF INTEREST

The authors declare no conflict of interest.

## DECLARATION OF TRANSPARENCY AND SCIENTIFIC RIGOUR

This Declaration acknowledges that this paper adheres to the principles for transparent reporting and scientific rigour of preclinical research as stated in the *BJP* guidelines for Design & Analysis, and Animal Experimentation, and as recommended by funding agencies, publishers, and other organizations engaged with supporting research.

## Supporting information


**Movie S1.** A representative behavior of the mice following THC withdrawal

**Figure S1.** SGIP1^−/−^ mice generation and characterization. Schematic depiction of the targeting construct used for homologous recombination of the SGIP1 gene. FRT sites allow enzymatic removal of the sequences used for selection, while two loxP sites flank exon 2. Removal of the critical exon 2 leads to loss of SGIP1 protein expression from the targeted allele. Arrows indicate the PCR probe annealing sites. Primer A: aggcacagcatccttaggcacagc, Primer B: gaatgtatcagggaaggttcagcc, Primer C: tgaactgatggcgagctcagacc. (A). The enzymatic excision of exon 2 was confirmed by PCR. The PCR products of the analysis of DNA, extracted from tail biopsy of the parental heterozygous (SGIP1^+/−^) generation, and selected siblings used in the experiment; knock‐out (SGIP1^−/−^) and wild type (WT) mice (B). The absence of SGIP1 protein in SGIP1^−/−^ mice was confirmed by immunoblotting from the brain homogenates, separated on SDS‐PAGE, and detected with the anti‐SGIP1 antibody (C). The body weights of the animals were measured during the behavioral testing and no difference between SGIP1^−/−^ and WT animals was found (males: (D), females: (E). The data in panels D, E were analyzed by two‐way ANOVA with repeated measures and are presented as means ± SEM
Figure S2. Additional parameters of the open field (OF) test. The entire arena (A) and center (C) velocity and resting time (E) were not altered in SGIP1^−/−^ males. The SGIP1^−/−^ females moved throughout the open field arena and in its center more slowly than WT controls (B, D) and rested more frequently over the entire arena (F). When in the center, SGIP^−/−^ male mice rested more than WT mice (G), while there was no difference in female cohorts (H). For clarity, we show the raw data for times spent in the center of the open field arena. Male SGIP1^−/−^ mice spent more time in the center of the open field (I), we did not detect any significant differences between the female SGIP1^−/−^ and WT mice (J). Both male and female SGIP1^−/−^ mice exhibited similar numbers of freezing episodes in the open field test as the WT controls (males: K, females: L). SGIP1^−/−^ male mice showed higher incidence of rearing (M), and there was no difference in female cohorts (N) The data were analyzed by Mann–Whitney U test (B, F, G, I, J,) or by parametric test when the data were normally distributed (A, C‐E, H, K‐N) and are presented as means ± SEM. * p < 0.05
Figure S3. Additional parameters of the elevated plus maze (EPM) tests. The number of visits into open and closed arms of the EPM is not altered by the deletion of the SGIP1 gene (males: A, females: B). Similarly, the incidence of rearing behavior is comparable in SGIP1^−/−^ and WT mice (males: C, females: D). Both male and female SGIP1^−/−^ mice spent more time in the open arms of the EPM (males: E, females: F). The data were analyzed by Mann–Whitney U test (E) or by t‐test where the data were normally distributed (C, D, F). The numbers of visits in the EPM were analyzed by two‐way ANOVA (A, B) and are presented as means ± SEM. * p < 0.05
Figure S4. Comparison of WIN and morphine effects on antinociception in SGIP1^−/−^ and WT males. Latency to dose analyses. Ligand concentration required for a specific MPE in the SGIP1 KO mice divided by the ligand concentration needed to reach the same MPE in WT mice was plotted on the X‐axis, while the ratio of the MPEs for that dose of ligand in the WT mice is plotted on the Y axis. The lines for the two ligands were then fit by linear regression. The results of the linear regressions are y = 5.789x + 4.38 and y = 1.487x + 3.453 for WIN and morphine, respectively. The steeper slope of the WIN line is consistent with a synergistic‐like effect with SGIP1 deletion, morphine‐induced antinociception is additive‐like with SGIP1 absence
Table S1. Statistical analysis of behavior tests results presented in Figure 1. Results of parametric t‐tests (SA) and multiple t‐tests (PPI). SA, spontaneous alteration; PPI, pre‐pulse inhibition
Table S2. Statistical analysis of behavior test results presented in Figure 2 and Suppl. Figures 1–3. Results of parametric, nonparametric (Mann–Whitney) t‐tests and two‐way ANOVA. For the results of the Mann–Whitney test we show median values and U‐values, for the results of parametric tests for the data with normal distribution we show mean values
Table S3. Statistical analysis of the cannabinoid tetrad test results presented in Figure 3. Results of three‐way ANOVA analysis
Table S4. Statistical analysis of the withdrawal experiment results presented in Figure 3. Results of three‐way ANOVA (headshakes, paw shakes, scratching/grooming) and R based general linear model (jumps). In order to analyze data with equal variances the headshakes and paw shakes data were log transformed
Table S5. Statistical analysis of tail flick tests results presented in Figure 4. The results of parametric t‐test, Mann–Whitney U test and two‐way ANOVA
Table S6. Statistical analysis of CB1R antagonist experiment results presented in Figure 5. The results of R based analysis by general linear model with a set of contrasts between defined groups
Table S7. Overview of used statistical tests

## Data Availability

The data that support the findings of this study are available from the corresponding author upon reasonable request. Some data may not be made available because of privacy or ethical restrictions.
