# Supplementary material for: SGIP1 is involved in regulation of emotionality, mood, and nociception and modulates in vivo signalling of cannabinoid CB1 receptors
Source: Br J Pharmacol. 2021 Feb 27;178(7):1588–604. doi: 10.1111/bph.15383 (PMC8795748; doi:10.1111/bph.15383)
Supplement: Supplementary file 2 — Figure S1. SGIP1−/− mice generation and characterization. Schematic depiction of the targeting construct used for homologous recombination of the SGIP1 gene. FRT sites allow enzymatic removal of the sequences used for selection, while two loxP sites flank exon 2. Removal of the critical exon 2 leads to loss of SGIP1 protein expression from the targeted allele. Arrows indicate the PCR probe annealing sites. Primer A: aggcacagcatccttaggcacagc, Primer B: gaatgtatcagggaaggttcagcc, Primer C: tgaactgatggcgagctcagacc. (A). The enzymatic excision of exon 2 was confirmed by PCR. The PCR products of the analysis of DNA, extracted from tail biopsy of the parental heterozygous (SGIP1+/−) generation, and selected siblings used in the experiment; knock‐out (SGIP1−/−) and wild type (WT) mice (B). The absence of SGIP1 protein in SGIP1−/− mice was confirmed by immunoblotting from the brain homogenates, separated on SDS‐PAGE, and detected with the anti‐SGIP1 antibody (C). The body weights of the animals were measured during the behavioral testing and no difference between SGIP1−/− and WT animals was found (males: (D), females: (E). The data in panels D, E were analyzed by two‐way ANOVA with repeated measures and are presented as means ± SEM Figure S2. Additional parameters of the open field (OF) test. The entire arena (A) and center (C) velocity and resting time (E) were not altered in SGIP1−/− males. The SGIP1−/− females moved throughout the open field arena and in its center more slowly than WT controls (B, D) and rested more frequently over the entire arena (F). When in the center, SGIP−/− male mice rested more than WT mice (G), while there was no difference in female cohorts (H). For clarity, we show the raw data for times spent in the center of the open field arena. Male SGIP1−/− mice spent more time in the center of the open field (I), we did not detect any significant differences between the female SGIP1−/− and WT mice (J). Both male and female SGIP1−/− mice exhibited similar [file BPH-178-1588-s002.pdf]

Supplemental information

Generation of SGIP1$^{-/-}$ mice

SGIP1$^{-/-}$ mice were generated in-house starting with embryonic stem cells obtained from the European Conditional Mouse Mutagenesis Program (EUCOMM). The cells (Sgip1$^{tm1a(EUCOMM)Hmgu}$) carried a SGIP1 gene (GeneBank Accession: NM_001285852) modified by homologous recombination. The ES cells were of C57Bl/NCrl background.

In these cells, the FRT sites flanked exon 2 of the SGIP1 gene and the LoxP sites bordered additional sequences (Suppl. Fig. 1A). Using a laser-assisted technique, ES cells were injected into 8-cell stage embryos to generate chimeric mice. Sgip1$^{tm1a+/-}$ mice were crossed. Selected offspring were mated with Flp-expressing Gt(ROSA)26Sor$^{tm2(CAG-flpo,-EYFP)Ics}$, to delete aberrant sequences, and their offspring were further crossed with a Cre-recombinase expressing strain Gt(ROSA)26Sor$^{tm1(ACTB-cre,-EGFP)Ics}$ to excise the Exon 2. Both mice were from the same source (Birling, Dierich et al., 2012). This resulted in a shift of the frame in the SGIP1 allele, causing global loss of SGIP1 protein in homozygous SGIP1$^{-/-}$ mice. The mice underwent more than 10 backcrossing to obtain constant C57Bl/NCrl background. This also led to the exclusion of the FLP and Cre recombinase coding sequences and to homogenization to C57Bl/NCrl background.

The ablation of SGIP1 was confirmed by PCR analysis and immunoblotting (Suppl. Fig. 1B and 1C).

Genotyping

Mice were genotyped using tail snip samples collected at the time of weaning. Tails were digested, genomic DNA isolated, and purified using the QIAGEN DNAeasy kit (QIAGEN) according to the manufacturer's instructions. After the extractions, 1.0 µg of DNA was amplified by Polymerase chain reaction (PCR), using combination of three primers designed to to identify both the wild-type and targeted alleles annealing inside and outside the deleted region.

Primer A: AGGCACAGCATCCTTAGGCACAGC,

Primer B: GAATGTATCAGGGAAGGTTCAGCC,

Primer C: TGAACTGATGGCGAGCTCAGACC

The A-B primer pair allowed amplification of a 456-bp band, that corresponds to the wild-type allele. The A-C primer pair allowed detection of a 245-bp band corresponding to the sequence of the targeted allele. For the PCR reaction we a polymerase without proofreading activity (Promega GoTaq® Green Master Mix). Denaturing, annealing, and extension steps were performed; initial step 95°C lasted 5 minutes, followed by 30 cycles of 95°C for 30 s, annealing at 60°C for 30 s, and extensions at 72°C for 2 minutes. The reaction was terminated at 72°C for 10 minutes, followed by 4°C till separation and visualization of the PCR products on 1.5% agarose gel.

Immunoblot analysis

Protein samples (50 µg of brain homogenate) were separated on SDS-PAGE Tris-glycine gel (10%) and transferred onto nitrocellulose membranes (Pall Corporation, USA). Membranes were blocked in 5% powdered milk, labeled with home-made guinea pig anti-SGIP1 antibody (H09/h23) 1:1000 characterized in our previous study (Hajkova et al., 2016) and secondary goat anti-guinea pig IgG-HRP antibody (Santa Cruz Biotechnology, USA) 1:5000.

Samples were visualized using the SuperSignal West FEMTO chemiluminescent substrate system (Thermo Fisher Scientific, USA) and detected on the LAS-300 system (Fujifilm, USA).

Supplemental Figures

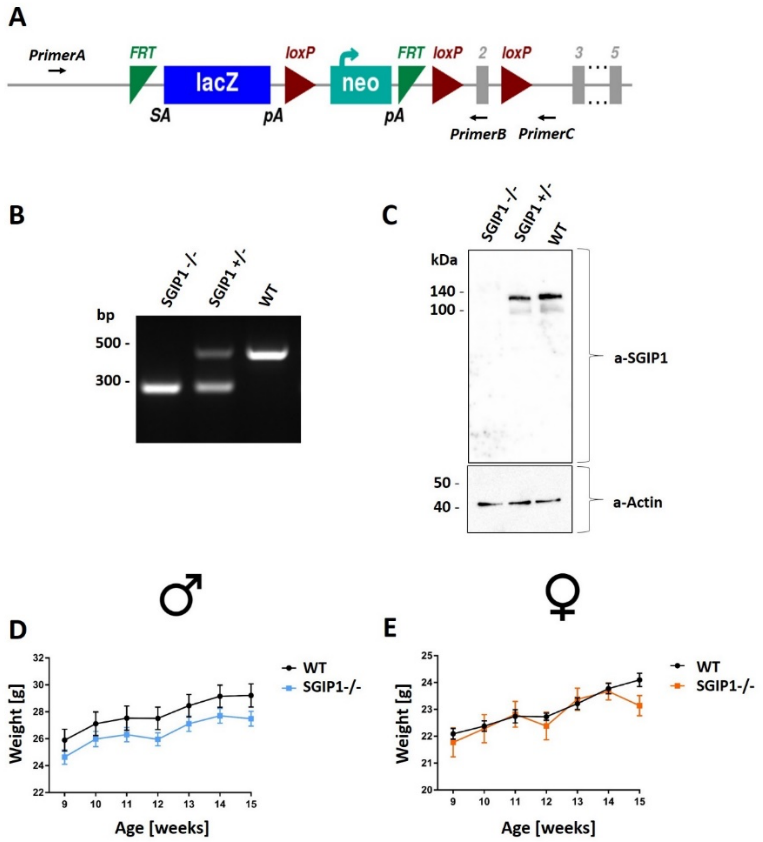

Supplemental figure 1. SGIP1$^{-/-}$ mice generation and characterization

Schematic depiction of the targeting construct used for homologous recombination of the SGIP1 gene. FRT sites allow enzymatic removal of the sequences used for selection, while two loxP sites flank exon 2. Removal of the critical exon 2 leads to loss of SGIP1 protein expression from the targeted allele. Arrows indicate the PCR probe annealing sites. Primer A: aggcacagcatccttaggcacagc, Primer B: gaatgtatcagggaaggttcagcc, Primer C: tgaactgatggcgagctcagacc. (A). The enzymatic excision of exon 2 was confirmed by PCR. The PCR products of the analysis of DNA, extracted from tail biopsy of the parental heterozygous (SGIP1$^{+/-}$) generation, and selected siblings used in the experiment; knock-out (SGIP1$^{-/-}$) and wild type (WT) mice (B). The absence of SGIP1 protein in SGIP1$^{-/-}$ mice was confirmed by immunoblotting from the brain homogenates, separated on SDS-PAGE, and detected with the anti-SGIP1 antibody (C). The body weights of the animals were measured during the behavioral testing and no difference between SGIP1$^{-/-}$ and WT animals was found (males: (D), females: (E). The data in panels D, E were analyzed by two-way ANOVA with repeated measures and are presented as means ± SEM

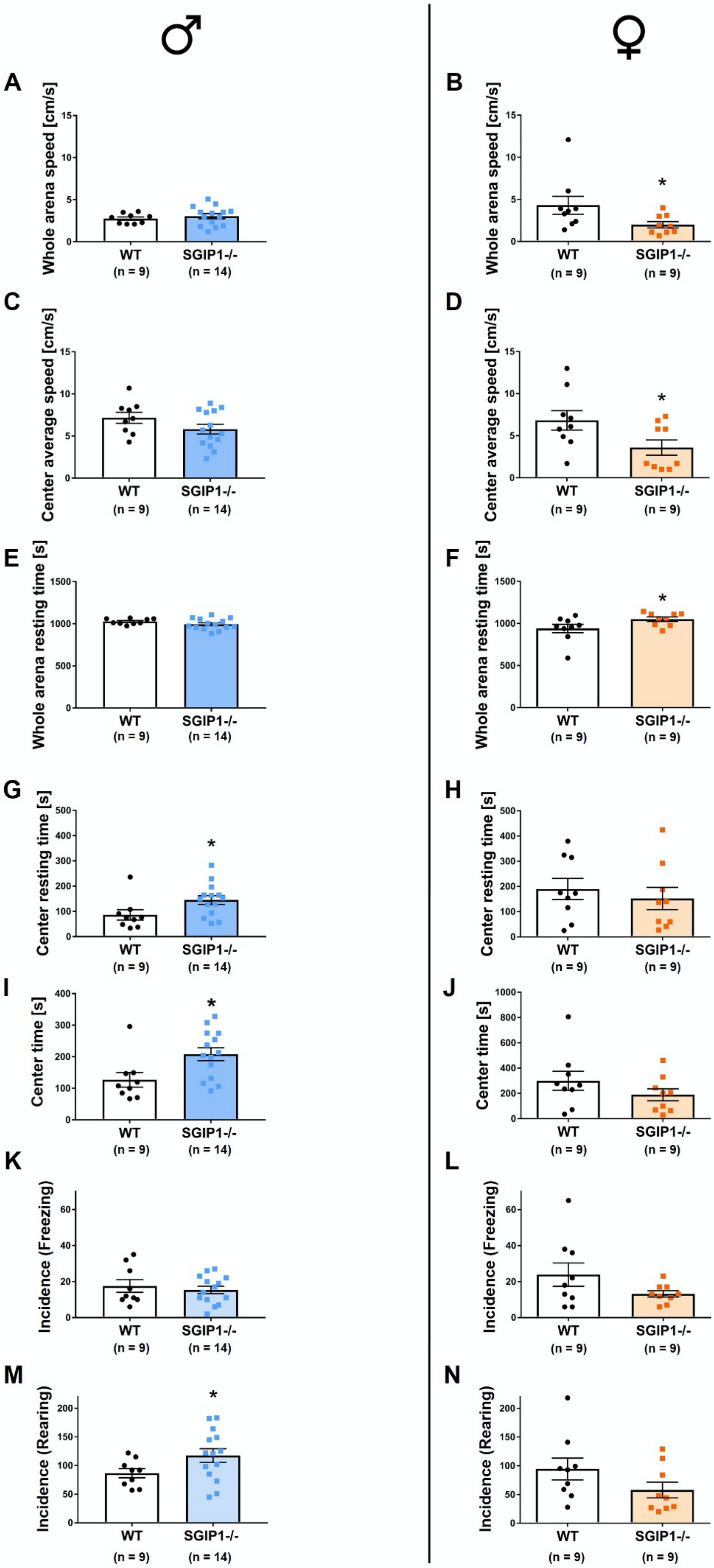
♂
♀
A
Whole arena speed [cm/s]
0
5
10
15
WT
(n = 9)
SGIP1-/-
(n = 14)
B
Whole arena speed [cm/s]
*
WT
(n = 9)
SGIP1-/-
(n = 9)
C
Center average speed [cm/s]
D
Center average speed [cm/s]
*
E
Whole arena resting time [s]
0
500
1000
1500
F
Whole arena resting time [s]
*
G
Center resting time [s]
0
100
200
300
400
500
*
H
Center resting time [s]
I
Center time [s]
*
J
Center time [s]
0
200
400
600
800
1000
K
Incidence (Freezing)
0
20
40
60
L
Incidence (Freezing)
M
Incidence (Rearing)
0
50
100
150
200
*
N
Incidence (Rearing)

Supplemental figure 2. Additional parameters of the open field (OF) test.

The entire arena (A) and center (C) velocity and resting time (E) were not altered in SGIP1$^{-/-}$ males. The SGIP1$^{-/-}$ females moved throughout the open field arena and in its center more slowly than WT controls (B, D) and rested more frequently over the entire arena (F). When in the center, SGIP$^{-/-}$ male mice rested more than WT mice (G), while there was no difference in female cohorts (H).

For clarity, we show the raw data for times spent in the center of the open field arena. Male SGIP1$^{-/-}$ mice spent more time in the center of the open field (I), we did not detect any significant differences between the female SGIP1$^{-/-}$ and WT mice (J). Both male and female SGIP1$^{-/-}$ mice exhibited similar numbers of freezing episodes in the open field test as the WT controls (males: K, females: L). SGIP1$^{-/-}$ male mice showed higher incidence of rearing (M), and there was no difference in female cohorts (N)

The data were analyzed by Mann-Whitney U test (B, F, G, I, J,) or by parametric test when the data were normally distributed (A, C-E, H, K-N) and are presented as means ± SEM. * $p<0.05$

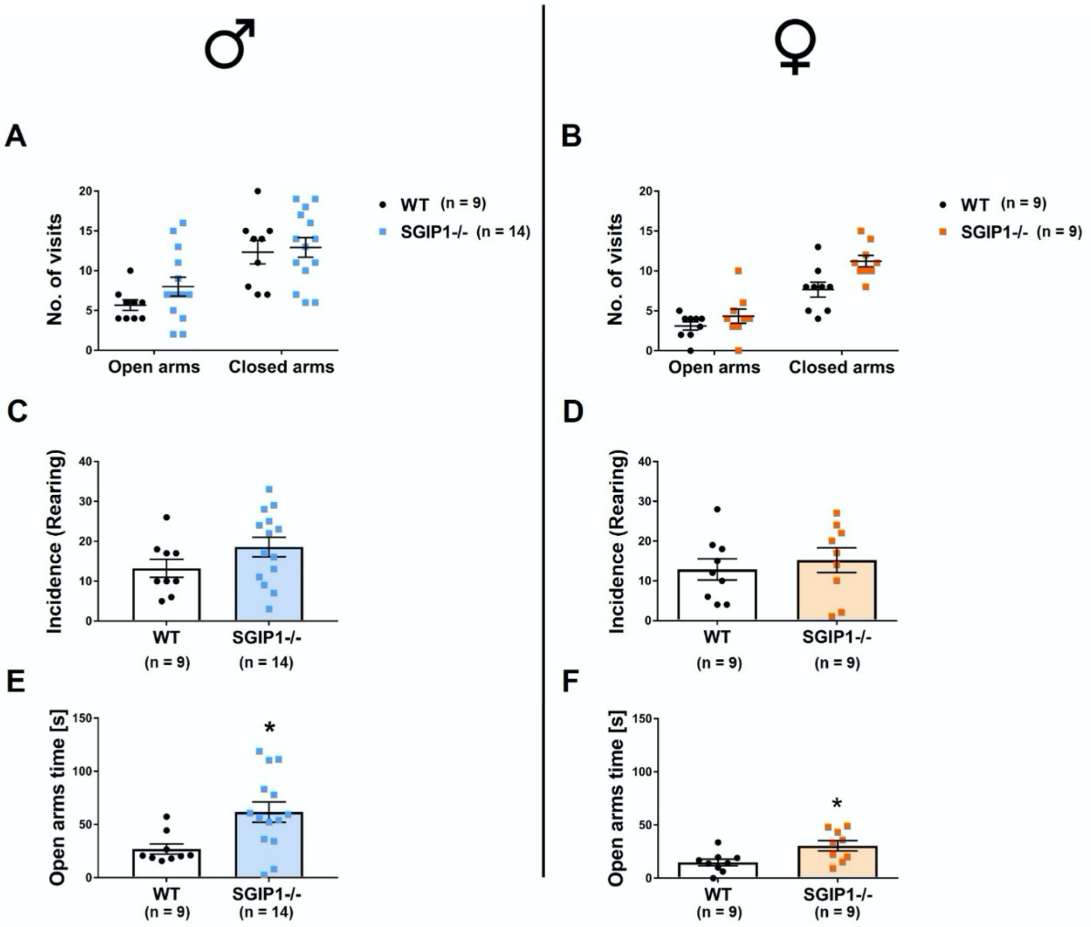

Supplemental figure 3. Additional parameters of the elevated plus maze (EPM) tests.

The number of visits into open and closed arms of the EPM is not altered by the deletion of the SGIP1 gene (males: A, females: B). Similarly, the incidence of rearing behavior is comparable in SGIP1$^{-/-}$ and WT mice (males: C, females: D). Both male and female SGIP1$^{-/-}$ mice spent more time in the open arms of the EPM (males: E, females: F).

The data were analyzed by Mann-Whitney U test (E) or by t-test where the data were normally distributed (C, D, F). The numbers of visits in the EPM were analyzed by two-way ANOVA (A, B) and are presented as means ± SEM. * $p<0.05$.

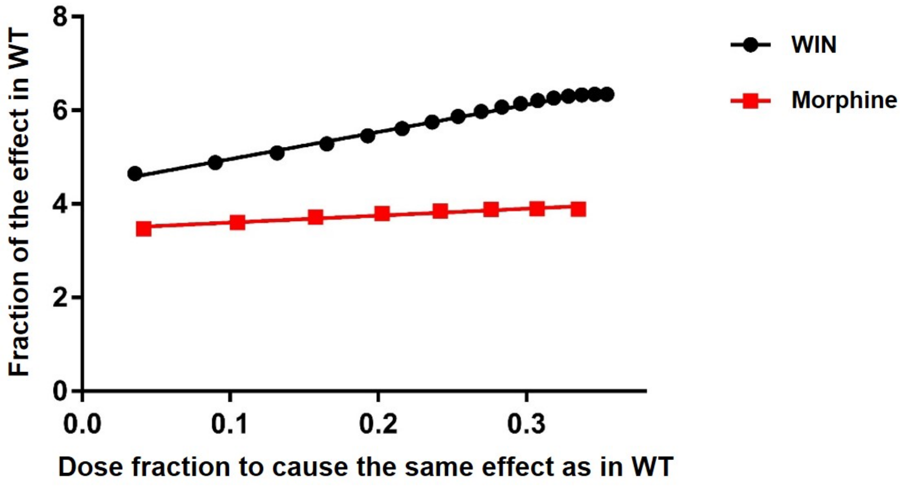

Supplemental figure 4. Comparison of WIN and morphine effects on antinociception in SGIP1$^{-/-}$ and WT males.

Latency to dose analyses. Ligand concentration required for a specific MPE in the SGIP1 KO mice divided by the ligand concentration needed to reach the same MPE in WT mice was plotted on the X-axis, while the ratio of the MPEs for that dose of ligand in the WT mice is plotted on the Y axis. The lines for the two ligands were then fit by linear regression. The results of the linear regressions are y=5.789x+4.38 and y=1.487x+3.453 for WIN and morphine, respectively. The steeper slope of the WIN line is consistent with a synergistic-like effect with SGIP1 deletion, morphine-induced antinociception is additive-like with SGIP1 absence.

Supplemental video. A representative behaviors of the mice following THC withdrawal.
$SGIP1^{-/-}$ and WT males were treated with intraperitoneal injections of THC (10 mg/kg) for 9 consecutive days. The mice were injected with a vehicle 30 min after the last drug deliveries, and after another 30 minutes CB1R inverse agonist rimonabant (10 mg/kg) was intraperitoneally injected, as illustrated in (Fig. 3E).

The sample video starts 25 minutes after the rimonabant injections.
Left chamber: $SGIP1^{-/-}$ male treated with THC.
Center chamber: $SGIP1^{-/-}$ male chronically treated with vehicle.
Right chamber: WT male chronically treated with THC.

Parametric tests

	Sex	Mean, n				P value
		WT	SGIP1$^{-/-}$			
Y-maze, % of SA	M	62.03, n=9	59.11, n=14			0.4533
	F	70.34, n=9	56.36, n=9			0.1195

Multiple t-tests

	Prepulse intensity [db]	Mean, n		t ratio	df	P value
		WT	SGIP1$^{-/-}$			
PPI MALES, % of PPI	70	29.85	38.11	1.14	21	0.4754
	77	40.32	58.08	2.645	21	0.0592
	82	49.31	59.44	1.343	21	0.4754
	85	53.91	62.56	1.101	21	0.4754
PPI FEMALES, % of PPI	70	30.78	32.98	0.3213	16	0.9526
	77	47.34	45.74	0.2497	16	0.9526
	82	51.42	61.47	2.683	16	0.0637
	85	54.33	56.68	0.4796	16	0.9526

Supplemental table 1. Statistical analysis of behavior tests results presented in Fig. 1.

Results of parametric t-tests (SA) and multiple t-tests (PPI). SA, spontaneous alteration; PPI, pre-pulse inhibition

Parametric tests and Mann–Whitney U tests

	Sex	Median, n/ mean, n		U value	P value
		WT	SGIP1$^{-/-}$		
OF, center time [%]	M	9.025, n=9	17.41, n=14	26	0.0191
	F	18.39, n=9	15.79, n=9		0.6355
OF, whole arena distance [cm]	M	3286, n=9	3666, n=14		0.3982
	F	3854, n=9	2379, n=9		0.0561
OF, whole arena speed [cm/s]	M	2.756, n=9	3.05, n=14		0.4289
	F	3.6, n=9	1.8, n=9	15	0.0228
OF, center speed [cm/s]	M	7.178, n=9	5.821, n=14		0.1362
	F	6.833, n=9	3.6, n=9		0.0431
OF, whole arena resting time [s]	M	1028, n=9	994.5, n=14		0.1226
	F	968.4, n=9	1060, n=9	16.5	0.0334
OF, center resting time [s]	M	75.7, n=9	151.8, n=14	28	0.0275
	F	190.1, n=9	152.4, n=9		0.5431
OF, center permanence time [s]	M	108.3, n=9	208.9, n=14	26	0.0191
	F	265.3, n=9	201.8, n=9	26	0.2224
OF, freezing episodes	M	17.56, n=9	15.36, n=14		0.6023

	F	23.89, n=9	13.22, n=9		0.1446
OF, rearing incidence	M	86.56, n=9	117.5, n=14		0.0421
	F	94.44, n=9	57.78, n=9		0.1378
EPM, open arms time [%]	M	6.89, n=9	19.18, n=14	25	0.0158
	F	4.944, n=9	10.16, n=9		0.0173
EPM, total distance [cm]	M	1275, n=9	1526, n=14		0.0322
	F	1437, n=9	1792, n=9		0.0146
EPM, rearing	M	13.22, n=9	18.57, n=14		0.1217
	F	12.89, n=9	15.22, n=9		0.5764
EPM, open arms time [s]	M	20.65, n=9	57.49, n=14	25	0.0158
	F	14.83, n=9	30.49, n=9		0.0173
LDB, light side time [%]	M	6.567, n=9	11.63, n=14	36	0.0956
	F	1.467, n=9	4.167, n=9	29	0.3284
LDB, total distance [cm]	M	980.8, n=9	1109, n=14		0.5433
	F	745, n=9	1087, n=9		0.1014
TST, immobility [s]	M	84.92, n=9	51.78, n=14		0.0334
	F	150.3, n=9	92.94, n=9		0.0013

Two-way ANOVA					Bonferroni's multiple comparisons test				
	Sex	Source of Variation	F (DFn, DFd)	P value	X axis factor	Mean 1	Mean 2	95.00% CI of diff.	P value
Weights [g]	M	Interaction	F (6, 126) = 1.17	0.3277					
		Weeks	F (6, 126) = 141.30	<0.0001					
		Genotype	F (1, 21) = 2.16	0.1564					
	F	Interaction	F (6, 114) = 2.87	0.0121					
		Weeks	F (6, 114) = 37.73	<0.0001					
		Genotype	F (1, 19) = 0.26	0.6167					
EPM, visits	M	Interaction	F (1, 21) = 2.40	0.1364					
		Arms	F (1, 21) = 106.70	<0.0001					
		Genotype	F (1, 21) = 0.77	0.3901					
	F	Interaction	F (1, 16) = 3.40	0.0836					
		Arms	F (1, 16) = 81.92	<0.0001					
		Genotype	F (1, 16) = 6.91	0.0182					
FC, freezing time [%]	M	Interaction	F (2, 50) = 0.35	0.7099					
		Context, Cue	F (2, 50) = 39.89	<0.0001					
		Genotype	F (1, 25) = 0.49	0.4926					
	F	Interaction	F (2, 50) = 0.41	0.6656					
		Context Cue	F (2, 50) = 36.64	<0.0001					

		Genotype	F (1, 25) = 1.21	0.2823						
FE, freezing time [log(s)]	M	Interaction	F (7, 154) = 0.24	0.9760						
		Days	F (7, 154) = 7.77	<0.0001						
		Genotype	F (1, 22) = 0.88	0.3586						
FE, freezing time [%]	F	Interaction	F (4, 88) = 4.05	0.0047	Day 1	30.39	30.05	-19.76 20.44	to	>0.9999
		Days	F (4, 88) = 8.40	<0.0001	Day 2	57.42	22.68	14.64 54.85	to	<0.0001
		Genotype	F (1, 22) = 12.77	0.0017	Day 3	35.17	14.83	0.24 40.44	to	0.0459
					Day 4	30.90	9.58	1.22 41.43	to	0.0319
					Day 5	27.61	8.15	-0.65 39.56	to	0.0630

Supplemental table 2. Statistical analysis of behavior test results presented in Fig. 2 and Suppl. Fig 1-3. Results of parametric, nonparametric (Mann-Whitney) t-tests and two-way ANOVA. For the results of the Mann-Whitney test we show median values and U-values, for the results of parametric tests for the data with normal distribution we show mean values.

Cannabinoid tetrad test

Three-way ANOVA				Bonferroni's multiple comparisons test				
	Source of Variation	F (DFn, DFd)	P value	X axis factor	Mean 1	Mean 2	95.00% CI of diff.	P value
Ring test %MPE	Days	F (2, 64) = 43.56	<0.0001	Day 1				
	Genotype	F (1, 32) = 14.52	0.0006	WT_VEH vs. WT_THC	-1.40	72.95	-88.94 to -59.76	<0.0001
	THC or VEH	F (1, 32) = 649.10	<0.0001	WT_VEH vs. SGIP1$^{-/-}$_VEH	-1.40	1.88	-17.88 to 11.31	>0.9999
	Days x Genotype	F (2, 64) = 4.71	0.0123	WT_THC vs. SGIP1-/-_THC	72.95	75.22	-16.86 to 12.32	>0.9999
	Days x THC or VEH	F (2, 64) = 42.32	<0.0001	SGIP1$^{-/-}$_VEH vs. SGIP1$^{-/-}$_THC	1.88	75.22	-87.93 to -58.74	<0.0001
	Genotype x THC or VEH	F (1, 32) = 10.21	0.0031	Day 4				
	Days x Genotype x THC or VEH	F (2, 64) = 7.75	0.0010	WT_VEH vs. WT_THC	-0.29	42.32	-57.20 to -28.02	<0.0001
				WT_VEH vs. SGIP1$^{-/-}$_VEH	-0.29	0.55	-14.60 to 14.58	>0.9999
				WT_THC vs. SGIP1-/-_THC	42.32	61.05	-33.32 to -4.137	0.0014
				SGIP1$^{-/-}$_VEH vs. SGIP1$^{-/-}$_THC	-0.28	61.05	-75.92 to -46.74	<0.0001
				Day 8				
				WT_VEH vs. WT_THC	-0.24	30.71	-45.55 to -16.37	<0.0001
				WT_VEH vs. SGIP1$^{-/-}$_VEH	-0.24	0.55	-15.38 to 13.80	>0.9999
				WT_THC vs. SGIP1-/-_THC	30.71	56.22	-40.10 to -10.92	<0.0001

				$SGIP1^{-/-}$_VEH vs. $SGIP1^{-/-}$_THC	0.55	56.22	-70.27 to -41.08	<0.0001
Tail flick %MPE	Days	F (2, 64) = 13.29	<0.0001	Day 1				
	Genotype	F (1, 32) = 5.31	0.0278	WT_VEH vs. WT_THC	-2.43	14.36	-30.77 to -2.82	0.0042
	THC or VEH	F (1, 32) = 71.56	<0.0001	WT_VEH vs. $SGIP1^{-/-}$_VEH	-2.43	-1.70	-14.70 to 13.24	>0.9999
	Days x Genotype	F (2, 64) = 1.65	0.1994	WT_THC vs. SGIP1-/-_THC	14.36	29.91	-29.52 to -1.57	0.0130
	Days x THC or VEH	F (2, 64) = 6.56	0.0026	$SGIP1^{-/-}$_VEH vs. $SGIP1^{-/-}$_THC	-1.70	29.91	-45.58 to -17.64	<0.0001
	Genotype x THC or VEH	F (1, 32) = 4.56	0.0406	Day 4				
	Days x Genotype x THC or VEH	F (2, 64) = 1.80	0.1740	WT_VEH vs. WT_THC	-4.20	8.62	-26.80 to 1.15	0.1256
				WT_VEH vs. $SGIP1^{-/-}$_VEH	-4.20	-3.03	-15.15 to 12.80	>0.9999
				WT_THC vs. SGIP1-/-_THC	8.62	11.41	-16.76 to 11.19	>0.9999
				$SGIP1^{-/-}$_VEH vs. $SGIP1^{-/-}$_THC	-3.03	11.41	-28.41 to -0.46	0.0340
				Day 8				
				WT_VEH vs. WT_THC	-4.47	3.84	-22.28 to 5.67	>0.9999
				WT_VEH vs. $SGIP1^{-/-}$_VEH	-4.47	-5.35	-13.09 to 14.85	>0.9999
				WT_THC vs. SGIP1-/-_THC	3.84	12.12	-22.26 to 5.69	>0.9999
				$SGIP1^{-/-}$_VEH vs. $SGIP1^{-/-}$_THC	-5.35	12.12	-31.44 to -3.50	0.0022

Temperature % change	Days	F (2, 64) = 15.39	<0.0001	Day 1				
	Genotype	F (1, 32) = 4.51	0.0415	WT_VEH vs. WT_THC	1.51	-4.89	2.81 to 10.00	<0.0001
	THC or VEH	F (1, 32) = 45.72	<0.0001	WT_VEH vs. SGIP1$^{-/-}$_VEH	1.51	0.74	-2.83 to 4.37	>0.9999
	Days x Genotype	F (2, 64) = 2.20	0.1194	WT_THC vs. SGIP1-/-_THC	-4.89	-0.95	-0.16 to 7.03	0.0837
	Days x THC or VEH	F (2, 64) = 33.53	<0.0001	SGIP1$^{-/-}$_VEH vs. SGIP1$^{-/-}$_THC	0.74	-0.95	5.48 to 12.67	<0.0001
	Genotype x THC or VEH	F (1, 32) = 1.00	0.324	Day 4				
	Days x Genotype x THC or VEH	F (2, 64) = 1.23	0.2986	WT_VEH vs. WT_THC	0.04	-0.43	-3.13 to 4.06	>0.9999
				WT_VEH vs. SGIP1$^{-/-}$_VEH	0.04	-0.83	-2.73 to 4.47	>0.9999
				WT_THC vs. SGIP1-/-_THC	-0.43	-0.95	-3.07 to 4.12	>0.9999
				SGIP1$^{-/-}$_VEH vs. SGIP1$^{-/-}$_THC	-0.83	-0.95	-3.48 to 3.72	>0.9999
				Day 8				
				WT_VEH vs. WT_THC	0.28	-0.56	-2.76 to 4.43	>0.9999
				WT_VEH vs. SGIP1$^{-/-}$_VEH	0.28	0.41	-3.73 to 3.46	>0.9999
				WT_THC vs. SGIP1-/-_THC	-0.56	-0.78	-3.37 to 3.82	>0.9999
				SGIP1$^{-/-}$_VEH vs. SGIP1$^{-/-}$_THC	0.41	-0.78	-2.40 to 4.79	>0.9999
Rotarod [s]	Days	F (3, 96) = 6.01	0.0009					
	Genotype	F (1, 32) = 2.24	0.1440					

THC or VEH	F (1, 32) = 4.31	0.0460
Days x Genotype	F (3, 96) = 1.16	0.3287
Days x THC or VEH	F (3, 96) = 4.43	0.0058
Genotype x THC or VEH	F (1, 32) = 3.21	0.0827
Days x Genotype x THC or VEH	F (3, 96) = 1.08	0.3632

Supplemental table 3. Statistical analysis of the cannabinoid tetrad test results presented in Fig. 3. Results of three-way ANOVA analysis.

THC withdrawal test

Three-way ANOVA				Bonferroni's multiple comparisons test				
	Source of Variation	F (DFn, DFd)	P value	X axis factor	Mean 1	Mean 2	95.00% CI of diff.	P value
Headshakes (log transformation)	Withdrawal treatment	F (2, 64) = 17,80	<0.0001	THC				
	Genotype	F (1, 32) = 0,9613	0.3342	WT_VEH vs. WT_THC	4.20	3.74	-0.98 to 1.89	>0.9999
	THC or VEH	F (1, 32) = 13,47	0.0009	SGIP1$^{-/-}$_VEH vs. SGIP1$^{-/-}$_THC	4.58	3.63	-0.48 to 2.39	>0.9999
	Withdrawal treatment x Genotype	F (2, 64) = 0,2308	0.7946	VEH				
	Withdrawal treatment x THC or VEH	F (2, 64) = 27,40	<0.0001	WT_VEH vs. WT_THC	4.00	2.08	0.48 to 3.36	0.0007
	Genotype x THC or VEH	F (1, 32) = 0,0006585	0.9797	SGIP1$^{-/-}$_VEH vs. SGIP1$^{-/-}$_THC	4.49	2.27	0.79 to 3.67	<0.0001
	Withdrawal treatment x Genotype x THC or VEH	F (2, 64) = 2,606	0.0817	Rimonabant				
				WT_VEH vs. WT_THC	4.01	3.87	-1.30 to 1.58	>0.9999
				SGIP1$^{-/-}$_VEH vs. SGIP1$^{-/-}$_THC	3.77	4.48	-2.15 to 0.73	>0.9999
				WT_VEH				

				THC vs. VEH	4.20	4.00	-0.93 to 1.33	>0.9999
				THC vs. rimonabant	4.20	4.01	-0.95 to 1.32	>0.9999
				VEH vs. rimonabant	4.00	4.01	-1.15 to 1.12	>0.9999
				WT_THC				
				THC vs. VEH	3.74	2.08	0.53 to 2.80	0.0001
				THC vs. rimonabant	3.74	3.87	-1.26 to 1.00	>0.9999
				VEH vs. rimonabant	2.08	3.87	-2.93 to -0.66	<0.0001
				SGIP1-/-_VEH				
				THC vs. VEH	4.58	4.49	-1.04 to 1.22	>0.9999
				THC vs. rimonabant	4.58	3.77	-0.32 to 1.94	0.8897
				VEH vs. rimonabant	4.49	3.77	-0.41 to 1.90	>0.9999
				SGIP1-/-_THC				
				THC vs. VEH	3.63	2.27	0.23 to 2.50	0.0047
				THC vs. rimonabant	3.63	4.48	-1.99 to 0.28	0.6334
				VEH vs. rimonabant	2.27	4.48	-3.34 to -1.08	<0.0001
Paw shakes (log transformation)	Withdrawal treatment	F (2, 64) = 69.02	<0.0001	THC				
	Genotype	F (1, 32) = 5.56	0.0247	WT_VEH vs. WT_THC	3.64	2.74	-0.59 to 2.39	>0.9999
	THC or VEH	F (1, 32) = 18.92	0.0001	WT_VEH vs. $SGIP1^{-/-}$_VEH	3.64	3.35	-1.20 to 1.79	>0.9999

Withdrawal treatment x Genotype	F (2, 64) = 3.88	0.0258	WT_THC vs. SGIP1-/-_THC	2.74	1.56	-0.31 to 2.67	0.4730
Withdrawal treatment x THC or VEH	F (2, 64) = 39.72	<0.0001	SGIP1$^{-/-}$_VEH vs. SGIP1$^{-/-}$_THC	3.35	1.56	0.29 to 3.28	0.0045
Genotype x THC or VEH	F (1, 32) = 1.55	0.2224	VEH				
Withdrawal treatment x Genotype x THC or VEH	F (2, 64) = 2.65	0.0781	WT_VEH vs. WT_THC	3.52	1.85	0.18 to 3.16	0.0122
			WT_VEH vs. SGIP1$^{-/-}$_VEH	3.52	3.17	-1.14 to 1.84	>0.9999
			WT_THC vs. SGIP1-/-_THC	1.85	0.38	-0.02 to 2.97	0.0577
			SGIP1$^{-/-}$_VEH vs. SGIP1$^{-/-}$_THC	3.17	0.38	1.30 to 4.29	<0.0001
			Rimonabant				
			WT_VEH vs. WT_THC	3.84	4.01	-1.66 to 1.32	>0.9999
			WT_VEH vs. SGIP1$^{-/-}$_VEH	3.84	3.63	-1.28 to 1.71	>0.9999
			WT_THC vs. SGIP1-/-_THC	4.01	3.88	-1.36 to 1.62	>0.9999
			SGIP1$^{-/-}$_VEH vs. SGIP1$^{-/-}$_THC	3.63	3.88	-1.75 to 1.24	>0.9999

				WT_VEH				
				THC vs. VEH	3.64	3.52	-0.86 to 1.10	>0.9999
				THC vs. rimonabant	3.64	3.84	-1.19 to 0.78	>0.9999
				VEH vs. rimonabant	3.52	3.84	-1.30 to 0.66	>0.9999
				WT_THC				
				THC vs. VEH	2.74	1.85	-0.10 to 1.87	0.1450
				THC vs. rimonabant	2.74	4.01	-2.26 to -0.29	0.0014
				VEH vs. rimonabant	1.85	4.01	-0.02 to 2.97	0.0577
				SGIP1-/-_VEH				
				THC vs. VEH	3.35	3.17	-0.81 to 1.16	>0.9999
				THC vs. rimonabant	3.35	3.63	-1.27 to 0.70	>0.9999
				VEH vs. rimonabant	3.17	3.63	-1.44 to 0.52	>0.9999
				SGIP1-/-_THC				
				THC vs. VEH	1.56	0.38	0.20 to 2.16	0.0046
				THC vs. rimonabant	1.56	3.88	-3.31 to -1.34	<0.0001
				VEH vs. rimonabant	0.38	3.88	-4.49 to -2.52	<0.0001
Scratching/ grooming	Withdrawal treatment	F (2, 64) = 21.63	<0.0001	THC				
	Genotype	F (1, 32) = 1.97	0.1696	WT_VEH vs. WT_THC	49.89	31.44	-18.76 to 55.65	>0.9999
	THC or VEH	F (1, 32) = 43.32	<0.0001	$SGIP1^{-/-}$_VEH vs. $SGIP1^{-/-}$_THC	51.67	10.11	4.35 to 78.76	0.0124

Withdrawal treatment x Genotype	F (2, 64) = 0.07	0.9341	VEH				
Withdrawal treatment x THC or VEH	F (2, 64) = 1.20	0.3079	WT_VEH vs. WT_THC	49.56	13.78	-1.43 to 73.00	0.0774
Genotype x THC or VEH	F (1, 32) = 0.61	0.4416	$SGIP1^{-/-}$_VEH vs. $SGIP1^{-/-}$_THC	45.44	3.00	5.24 to 79.65	0.0092
Withdrawal treatment x Genotype x THC or VEH	F (2, 64) = 1.18	0.3127	Rimonabant				
			WT_VEH vs. WT_THC	81.22	36.56	7.46 to 81.87	0.0043
			$SGIP1^{-/-}$_VEH vs. $SGIP1^{-/-}$_THC	72.89	31.44	4.24 to 78.65	0.0128
			WT_VEH				
			THC vs. VEH	49.89	49.56	-30.25 to 30.91	>0.9999
			THC vs. rimonabant	49.89	81.22	-61.91 to -0.75	0.0379
			VEH vs. rimonabant	49.56	81.22	-62.25 to -1.09	0.0335
			WT_THC				
			THC vs. VEH	31.44	13.78	-12.91 to 48.25	>0.9999
			THC vs. rimonabant	31.44	36.56	-35.69 to 25.47	>0.9999
			VEH vs. rimonabant	13.78	36.56	-26.43 to 47.98	>0.9999
			SGIP1-/-_VEH				

	THC vs. VEH	51.67	45.44	-24.36 to 36.80	>0.9999
	THC vs. rimonabant	51.67	72.89	-51.80 to 9.36	>0.9999
	VEH vs. rimonabant	45.44	72.89	-58.02 to 3.136	0.1522
	SGIP1-/-_THC				
	THC vs. VEH	10.11	3.00	-23.47 to 37.69	>0.9999
	THC vs. rimonabant	10.11	31.44	-51.91 to 9.25	>0.9999
	VEH vs. rimonabant	3.00	31.44	-59.02 to 2.14	0.1075
R based linear model					

	contrast	null.value	estimate	std.error	statistic	adj.p.value
Jumps						
	THC					
	WT_VEH vs. WT_THC	0	-1.44E-10	2858.766	-5.04E-14	>0.9999
	SGIP1$^{-/-}$_VEH vs. SGIP1$^{-/-}$_THC	0	16.79851	1905.844	0.008814	>0.9999
	WT_THC vs. SGIP1$^{-/-}$_THC	0	16.7985077	2021.452996	0.008310115	>0.9999
	VEH					
	WT_VEH vs. WT_THC	0	4.39E-13	2858.766	1.54E-16	>0.9999
	SGIP1$^{-/-}$_VEH vs. SGIP1$^{-/-}$_THC	0	19.36346	1905.844	0.01016	>0.9999
	WT_THC vs. SGIP1$^{-/-}$_THC	0	19.36345705	2021.452882	0.00957898	>0.9999
	Rimonabant					
	WT_VEH vs. WT_THC	0	20.62986	2021.453	0.010205	>0.9999
	SGIP1$^{-/-}$_VEH vs. SGIP1$^{-/-}$_THC	0	5.46806	1.002107	5.456561	<0.0001
	WT_THC vs. SGIP1$^{-/-}$_THC	0	0.943557858	0.128119212	7.364686723	<0.0001

WT_VEH					
THC vs. rimonabant	0	-1.45E-10	2858.766	-5.06E-14	>0.9999
THC vs. VEH	0	-1.45E-10	2858.766	-5.08E-14	>0.9999
WT_THC					
THC vs. rimonabant	0	20.62986	2021.453	0.010205	>0.9999
THC vs. VEH	0	-6.24E-13	2858.766	-2.18E-16	>0.9999
SGIP1-/-_VEH					
THC vs. rimonabant	0	16.10536	1905.844	0.008451	>0.9999
THC vs. VEH	0	-1.45E-13	2695.27	-5.39E-17	>0.9999
SGIP1-/-_THC					
THC vs. rimonabant	0	4.774913	0.710084	6.724433	<0.0001
THC vs. VEH	0	2.564949	0.733799	3.495437	0.0054

Supplemental table 4. Statistical analysis of the withdrawal experiment results presented in Fig. 3.
Results of three-way ANOVA (headshakes, paw shakes, scratching/grooming) and R based general linear model (jumps). In order to analyze data with equal variances the headshakes and paw shakes data were log transformed.

Parametric test and Mann–Whitney U test

	Sex	Median, n/ mean, n		U value	P value
		WT	SGIP1$^{-/-}$		
Tail flick, average latency	M	1.983, n=9	2.872, n=14	8	0.0002
	F	1.336, n=11	2.122, n=11		0.0032

Two-way ANOVA

	Sex	Source of Variation	F (DFn, DFd)	P value
Tail flick, single trial latency	M	Interaction	F (2, 42) = 0.0718	0.9309
		Trials	F (2, 42) = 0.0401	0.9608
		Genotype	F (1, 21) = 9.933	0.0048
	F	Interaction	F (2, 40) = 0.9584	0.3921
		Trials	F (2, 40) = 3.117	0.0552
		Genotype	F (1, 20) = 12.1	0.0024

Supplemental table 5. Statistical analysis of tail flick tests results presented in Fig. 4.

The results of parametric t-test, Mann-Whitney U test and two-way ANOVA.

R based linear model

	contrast	null.value	estimate	std.error	statistic	adj.p.value
Day	Baseline					
1	WT_RIM vs. WT_VEH	0	0.132593	0.357999	0.370372	>0.9999
	SGIP1-/-_RIM vs. SGIP1-/-_VEH	0	0.254815	0.357999	0.711776	>0.9999
	SGIP1-/-_RIM vs. WT_RIM	0	1.744074	0.357999	4.871733	0.0002
	SGIP1-/-_VEH vs. WT_VEH	0	1.621852	0.357999	4.530329	0.0002
	SGIP1-/- vs. WT	0	3.862963	0.253143	15.25998	<0.0001
	RIM vs. VEH	0	3.995556	0.253143	15.78377	<0.0001
	30 min					
	WT_RIM vs. WT_VEH	0	-0.30444	0.357999	-0.85041	>0.9999
	SGIP1-/-_RIM vs. SGIP1-/-_VEH	0	-1.37444	0.357999	-3.83924	0.0046
	SGIP1-/-_RIM vs. WT_RIM	0	1.254444	0.357999	3.504047	0.0170
	SGIP1-/-_VEH vs. WT_VEH	0	2.324444	0.357999	6.492885	<0.0001
	SGIP1-/- vs. WT	0	3.969815	0.253143	15.68208	<0.0001
	RIM vs. VEH	0	1.559444	0.253143	6.160322	<0.0001
	60 min					
	WT_RIM vs. WT_VEH	0	0.057778	0.357999	0.161391	>0.9999
	SGIP1-/-_RIM vs. SGIP1-/-_VEH	0	-0.56556	0.357999	-1.57977	0.9290
	SGIP1-/-_RIM vs. WT_RIM	0	1.365556	0.357999	3.814414	0.0045
	SGIP1-/-_VEH vs. WT_VEH	0	1.988889	0.357999	5.555575	<0.0001
	SGIP1-/- vs. WT	0	4.081481	0.253143	16.1232	<0.0001
	RIM vs. VEH	0	4.056111	0.253143	16.02298	<0.0001
	WT					
	60 min vs Baseline	0	0.103704	0.170846	0.607001	>0.9999
	30 min vs Baseline	0	0.00037	0.170846	0.002168	>0.9999
	SGIP1-/-					
	60 min vs Baseline	0	-0.86759	0.215951	-4.01754	0.0023
	30 min vs Baseline	0	0.106852	0.170846	0.625427	>0.9999
Day 3	Baseline					
	WT_RIM vs. WT_VEH	0	0.417407	0.357999	1.165947	0.9970
	SGIP1-/-_RIM vs. SGIP1-/-_VEH	0	-0.15222	0.357999	-0.4252	>0.9999
	SGIP1-/-_RIM vs. WT_RIM	0	1.205926	0.357999	3.368519	0.0250
	SGIP1-/-_VEH vs. WT_VEH	0	1.775555	0.357999	4.95967	<0.0001
	SGIP1-/- vs. WT	0	3.462592	0.253143	13.67839	<0.0001
	RIM vs. VEH	0	2.707407	0.310036	8.732558	<0.0001
	30 min					
	WT_RIM vs. WT_VEH	0	0.145556	0.357999	0.406581	>0.9999

SGIP1-/-_RIM vs. SGIP1-/-_VEH	0	-0.61333	0.357999	-1.71323	0.8681
SGIP1-/-_RIM vs. WT_RIM	0	1.005556	0.357999	2.808824	0.1395
SGIP1-/-_VEH vs. WT_VEH	0	1.764444	0.357999	4.928633	<0.0001
SGIP1-/- vs. WT	0	1.909444	0.253143	7.542938	<0.0001
RIM vs. VEH	0	1.909444	0.253143	7.542938	<0.0001
60 min					
WT_RIM vs. WT_VEH	0	0.63	0.357999	1.759783	0.8424
SGIP1-/-_RIM vs. SGIP1-/-_VEH	0	0.142222	0.357999	0.39727	>0.9999
SGIP1-/-_RIM vs. WT_RIM	0	1.214444	0.357999	3.392315	0.0244
SGIP1-/-_VEH vs. WT_VEH	0	1.702222	0.357999	4.754827	<0.0001
SGIP1-/- vs. WT	0	2.339444	0.253143	9.24158	<0.0001
RIM vs. VEH	0	2.339444	0.253143	9.24158	<0.0001
WT					
60 min vs Baseline	0	0.087778	0.170846	0.513782	>0.9999
30 min vs Baseline	0	0.035556	0.170846	0.208114	>0.9999
SGIP1-/-					
60 min vs Baseline	0	0.05537	0.170846	0.324096	>0.9999
30 min vs Baseline	0	-0.07019	0.170846	-0.41081	>0.9999

Supplemental table 6. Statistical analysis of CB1R antagonist experiment results presented in Fig. 5.
The results of R based analysis by general linear model with a set of contrasts between defined groups.

Test	Data in the figures
Parametric t-test	1A-B, 2B-D, 2F-H, 2J, 2L-N, 4B, Suppl.2A, Suppl.2C-E, Suppl.2H, Suppl.2K-N, Suppl.3C-D, Suppl.3F
Mann-Whitney U-test	2A, 2E, 2I, 2K, 4A, Suppl.2B, Suppl.2F-G, Suppl.2I-J, Suppl.3E
Multiple t-tests	1C-D
Two-way ANOVA with repeated mesures	2O-R, 4C-D, Suppl.1D-E, Suppl.3A-B
Three-way ANOVA	3A-D, 3F-H
Nonlinear regression, ED_{50}	4E-J
General linear model	5A-B
General linear model, Poisson distribution	3I

Supplemental table 7. Overview of used statistical tests.
